# Protecting women's health in sport: the role of low energy availability in the Female Athlete Triad and RED-S

**DOI:** 10.3389/fspor.2026.1776533

**Published:** 2026-05-20

**Authors:** Hadeel Ali Ghazzawi, Batool Khataybeh, Raghad Al Aqaili, Lana Alnimer, Razan Mahmoud Omoush, Hebah Abdalla Ali

**Affiliations:** 1 Department of Nutrition and Food Technology, School of Agriculture, The University of Jordan, Amman, Jordan; 2 Department of Nutrition and Food Technology, Faculty of Agriculture, Jordan University of Science and Technology, Irbid, Jordan; 3 Independent Researcher, Mississauga, ON, Canada

**Keywords:** bone mineral density, Female Athlete Triad, low energy availability, menstrual dysfunction, Relative Energy Deficiency in Sport (RED-S)

## Abstract

**Background:**

The metabolic and physiological risks of the Female Athlete Triad and Relative Energy Deficiency in Sport (RED-S) remain widely under-recognized in athletic populations. Low Energy Availability (LEA), often driven by disordered eating (DE) and eating disorders (EDs), serves as the primary driver of multi-systemic impairments.

**Materials and methods:**

This narrative review synthesizes evidence from 151 studies identified via the Scopus database. The selection process involved a careful screening of 1,589 records, focusing on the interrelationships between energy availability, disordered eating, menstrual function, and bone mineral density across various female athletic populations.

**Results:**

The synthesis confirms that LEA leads to profound endocrine disruptions, in which weight stability often serves as a deceptive metabolic mask for underlying impairments. A significant correlation exists between disordered eating behaviors and the onset of LEA, creating a cycle that compromises hormonal integrity. Menstrual dysfunction serves as a vital clinical barometer; the resulting hypoestrogenism disrupts bone remodeling, leading to site-specific skeletal vulnerability—particularly in the lumbar spine. Furthermore, psychobehavioral factors, such as Body Image Overestimation, were identified as potent predictors of energy deficiency.

**Conclusion:**

Protecting the female athlete requires a proactive, multidisciplinary framework involving medical professionals, coaches, and families. Effective management must prioritize restoring energy balance to normalize menstrual function and safeguard long-term skeletal health. Future research should focus on targeted nutritional strategies and screening tools to detect early signs of LEA and disordered eating.

## 1 Introduction

Physical exercise is one of the most important factors promoting the overall health, preventing injuries, improving physical performance, psychological and social well-being of individuals (1–4). Such activity is especially important for women, given the physiological nature of their bodies and the changes they undergo. Exercise has become an absolute necessity for maintaining their physical and psychological health (5, 6). Society's perception of women's body shape often means that women consume significantly fewer calories than men, especially when it comes to carbohydrates (7). Given the high training loads and associated energy expenditure of female athletes, inadequate energy intake often disrupts reproductive cycles and negatively affects bone, muscle, brain, and immune health (8). Female athletes face different challenges and considerations when balancing adequate nutritional needs with energy expenditure during exercise (9). Energy availability (EA) is defined as the amount of energy available for body functions after subtracting the energy consumed during exercise from the energy consumed by the female athlete from meals. Taking into account the timing of meals in relation to exercise to improve training adaptations, performance, and athlete health across all phases of the menstrual cycle (9). A recent study by Sims et al. (9) reported that eumenorrheic female athletes should consume 0.32–0.38 g·kg^−1^ of high-quality protein before and after exercise to promote favorable adaptations, or alternatively ingest approximately 10 g of essential amino acids to reduce exercise-induced amino acid oxidation and meet increased amino acid demands. In several studies it was demonstrated that the Female Athlete Triad (TRIAD), a term first described in 1992 by the American College of Sports Medicine (ACSM), a prevalent problem in female athletes, is now recognized as a complex and interconnected set of three causes: low energy availability (LEA), menstrual dysfunction (amenorrhea), and low bone mineral density (BMD). Recent studies confirm the continued existence of these risks within the broader and more comprehensive concept of relative energy deficiency in sport (RED-S) (10–12). The root cause of this syndrome may be a lack of dietary energy intake in female athletes, which leads to an increased risk of fractures and long-term skeletal damage (13–15). Exercise itself may not directly cause amenorrhea, but it's the imbalance between energy intake and energy expenditure—known as LEA—that can lead to this (16). When a body starts expending a lot of energy through exercise and deprives itself of the energy it needs for its daily vital functions, it then conserves energy by shutting down systems it deems non-essential at that time, such as reproduction and growth, including bone building. Consequently, the brain reduces or suppresses the release of hormones that control ovulation (16–18). Furthermore, amenorrhea is also associated with decreased estrogen levels, which can be a cause of osteoporosis. A diet low in calcium and vitamin D may also contribute to reduced bone density (19). Eating disorders and disordered eating attitudes (DEAs) and LEA are also associated with an increased risk of primary dysmenorrhea and imbalances in cortisol and prolactin levels (20). These findings underscore the urgent need to use effective screening tools such as BEDA-Q and LEAF-Q for the early detection of these interrelated health risks, which include eating behaviors and physiological function, especially since awareness of the Triad/RED-S remains low even among competitive athletes (10, 21).

Women's sports are considered one of the most significant vital fields that reflect societal development and the growing role of women in achieving a balance between community participation and public health (5, 6). Female athletes can access energy balance information, but unfortunately, this information often lacks reliable scientific sources. Energy balance information for female athletes should include information about eating disorders, weight management, and awareness of body composition rather than just body weight. It should also include energy needs, macronutrient and micronutrient requirements, hydration, and supplementation (15, 22). Therefore, this review aims to provide a comprehensive and in-depth analysis of the complex relationship between Triad and eating disorders (EDs), with a particular focus on the physiological pathways linking low energy availability (LEA), hormonal imbalances, and bone health deterioration. The paper also seeks to highlight modern diagnostic tools and long-term health risks, such as BEDA-Q and LEAF-Q, to provide professionals with an evidence-based framework for the early identification and mitigation of these risks in female athletes.

## 2 Materials and methods

### 2.1 Data selection

**Figure 1:**
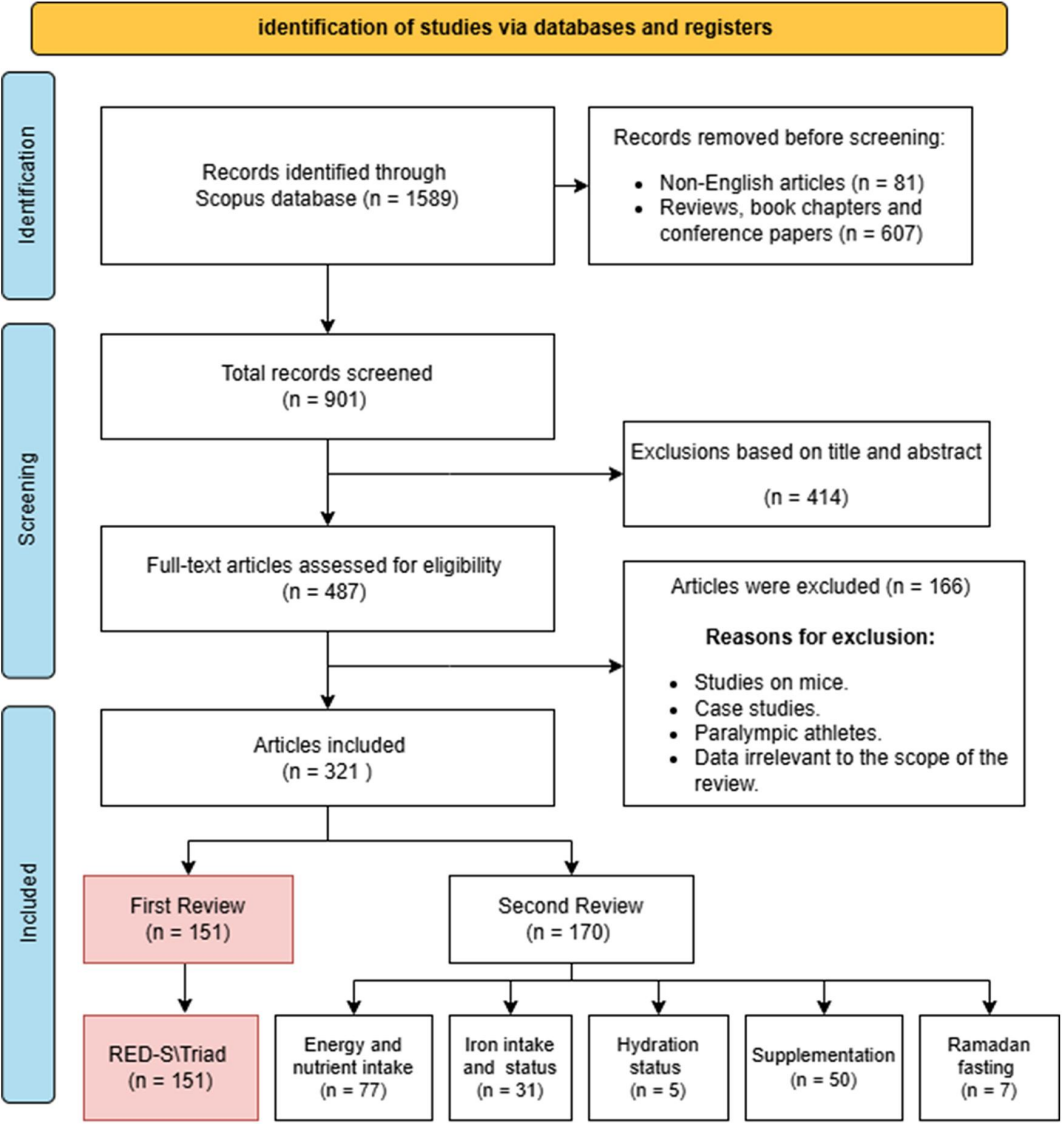
PRISMA diagram with the eligibility evaluation and data extraction procedure.

A comprehensive literature search was conducted using the Scopus database to identify studies examining nutrition, energy availability, and physiological outcomes in female athletes. The following query string in the Scopus advanced search: TITLE-ABS-KEY ((“female athlete*” OR “women athlete*”) AND (“energy availability” OR “energy deficiency” OR (“menstrual cycle” AND performance) OR “female athlete triad” OR [“bone health” AND (“calcium” OR “vitamin D”)] OR “macronutrient intake” OR “dietary intake”)). In total, 1,589 records were identified. After excluding 81 non-English publications and 607 review papers, book chapters, and conference proceedings, 901 articles remained for screening. Following the evaluation of titles and abstracts, 414 records were removed due to irrelevance to the research topic. The remaining 487 full-text articles were assessed for eligibility based on the following inclusion criteria: (1) studies involving female athletes only of any age, (2) articles published in English, and (3) studies published between 1991 and 2025. A total of 166 articles were excluded if they were conducted on male participants, animal models, or Paralympic athletes, were case studies, or presented data that did not align with the scope of the review. Ultimately, 321 articles met the inclusion criteria. The study selection process followed PRISMA guidelines (Figure 1).

### 2.2 Data extraction

Data from all included studies were independently extracted by two researchers using a standardized Excel-based extraction form. The form captured the following variables for each study: first author, year of publication, study type, sample size, participant age, primary outcomes, and the main focus of the study.

### 2.3 Data synthesis

Following data extraction, the 321 eligible studies were organized into two main comprehensive reviews to address different aspects of nutrition and performance in female athletes. The first review focused on the Female Athlete Triad (Triad)\RED-S, examining the interrelationships among low energy availability, disordered eating, menstrual dysfunction, and bone health. This review synthesized findings from 151 studies and included a total of 2,7103 female athletes. The second review included 170 studies and concentrated on energy intake, dietary macronutrient and micronutrient intake, iron deficiency, supplementation, hydration strategies, and nutritional intervention. These studies were analyzed to identify common dietary patterns, nutrient adequacy, and their impact on athletic performance, recovery, and overall health. Together, the two reviews provided a comprehensive overview of the nutritional challenges and physiological consequences associated with energy deficiency and dietary habits in female athletes.

## 3 Results

A total of 151 studies met the eligibility criteria and were included in the final synthesis. These studies examined the clinical and physiological dimensions of Relative Energy Deficiency in Sport (RED-S) and the Triad, with particular emphasis on low energy availability, disordered eating and eating disorders, menstrual function, and bone mineral density in female athletes. The key characteristics and principal findings of the included studies are summarized in Supplementary Table S1.

### 3.1 Low energy availability definition and screening tool

Low energy availability (LEA) serves as the central etiological factor underlying Relative Energy Deficiency in Sport (RED-S), a multisystemic syndrome that compromises both athletic performance and overall health (171, 172). According to the IOC, REDs is defined as a syndrome characterized by impaired physiological and/or psychological functioning resulting from prolonged or severe exposure to LEA, which ultimately compromises health (171). LEA, alongside menstrual dysfunction, and low bone mineral density are the core components of the Triad (171). LEA emerges when energy intake fails to support training demands and essential physiological functions (171, 173). Such deficiencies may lead to reproductive, endocrine, gastrointestinal, cardiovascular, and skeletal disturbances (171, 173). LEA is typically defined as an intake below the recommended threshold of 45 kcal/kg fat-free mass/day (68). The RED-S model extends the triad by integrating LEA, with or without ED, as the core determinant of both driving menstrual dysfunction and reduced BMD. The REDs model extends the triad by integrating LEA, with or without ED, as the core determinant of both driving menstrual dysfunction and reduced BMD.

The Low Energy Availability in Females Questionnaire (LEAF-Q) has been widely used as a screening tool for early identification of the Female Athlete Triad-related symptoms (53). Several studies have agreed that the LEAF-Q is a reliable tool for the identification of LEA risk and its associated physiological disturbances (55, 96, 99, 129, 170). On the other hand, other findings contradict its diagnostic usefulness. Dasa et al. and Rogers et al. (53, 125) reported that the LEAF-Q lacks specificity in female football players, concluding that it cannot accurately classify athletes as “high risk” of conditions related to LEA and should not be used as a surrogate diagnostic tool for LEA.

#### 3.1.1 Prevalence of LEA across different sport disciplines and team-based

Research has demonstrated LEA as a worldwide concern occurring across diverse sports, competitive levels, and age groups, in particular, high prevalence among female athletes. The prevalence of LEA varies considerably, reflecting differences in sport-specific demands and assessment methods. In a cross-sectional study of 112 female athletes, Sharps et al. (132) reported that 53% were classified as at risk of LEA using the LEAF-Q. Similarly, Muia et al. (106) identified clinically low energy availability in 17.9% of participants, while Hoch et al. (79) reported LEA in 36% of varsity athletes. More recently, Khatib et al. (86) documented a substantially higher prevalence, with 66.4% of female athletes affected.

#### 3.1.2 Low energy availability in runners and endurance

LEA appears to be particularly prevalent among female runners. Holsen Kyte et al. (91) reported a prevalence of 47% among competitive runners, whereas Karlsson et al. (84) observed a lower prevalence, with LEA symptoms identified in 19% of female runners aged 18–39 years (sixteen participants). Dervish et al. (57) observed a prevalence of 47.3% in UK female runners, while Sharp et al. (131) more recently reported that 64% of younger athletes (<35 years) and 29% of master's athletes (>35 years) were at significant risk of RED-S, with a median age of 32 years in the UK. Additionally, Wilwand et al. (168) examined non-competitive, recreationally active female runners aged 18–25 years and found that 82% of participants with two or more stress fractures were categorized as ‘at risk’ of LEA.

Additional evidence supports the findings from endurance sports. For example, Wasserfurth et al. (10) reported that 28% of German endurance athletes were classified as having relative energy deficiency in sport (RED-S), while 14% met the criteria for low energy availability based on the LEAF-Q (fourteen and two athletes, respectively). Earlier work by Melin et al. (101) demonstrated a substantially higher prevalence of LEA (63%) among female endurance athletes, with a mean age of 26.2 ± 5.5 years. Langa et al. (94) found LEA-related symptoms in 50% of female triathletes.

#### 3.1.3 Low energy availability in team sport

Regarding team sport, Reed et al. (119) conducted a study on 19 Division I female soccer players aged 18–21 years across the pre-, mid-, and post-seasons and reported low energy availability (530 kcal/kg FFM) in 26.3%, 33.3%, and 11.8% of participants, respectively. In another study conducted in NCAA Division I athletes or highly active women, Garay et al. (69) found that 63% of participants met criteria for LEA (<30 kcal/kg FFM/day). Moreover, in the Victorian Football League Women’s competition, Condo et al. (48) found that 30% of 30 players aged 18–35 years were at risk of LEA. Silvennoinen et al. (134) found that 41% (*n* = 72) of participants scored ≥8 points in the LEAF-Q among Finnish national to international-level female athletes indicating a high risk of problematic LEA.

Dasa et al. (52) found that 22% of 60 players from three Norwegian football teams, were at risk for RED-S, with 17% at mild, 3% at moderate-to-high, and 2% at very high/extreme risk. While in another study, Dasa et al. (53) reported that 32% of participants were classified as at risk according to the LEAF-Q. In professional female soccer, Saifi et al. (126) reported that 12% of 25 players aged 19–30 years had LEA according to LEAF-Q, whereas Dobrowolski and Włodarek (59) revealed that 20 of 31 participants across various league levels exhibited LEA. Among female youth football players aged 13–18 years, Łuszczki et al. (99) found that 64.7% were classified as being at-risk for the triad according to LEAF-Q scores, highlighting an alarming growing problem in this population.

Furthermore, all 21 professional female handball players included in the research with mean age of 22 ± 4 years, by Miralles-Amorós et al. (104) demonstrated LEA. Çetiner-Okşin et al. (43) reported that 80% of 15 Basketball players with mean age of 19.5 ± 1.3 years had negative energy balance, with 40% had low EA, and 46.7% had reduced EA. Consistently, among collegiate female volleyball players, according to Toews et al. (156) 21 athletes (mean age 20.3 ± 1.08 years) showed potential for LEA throughout the competitive season, with negative changes in nutritional status from pre-competition to competition phases regardless of living arrangements. In a sample of 122 adult Irish female Gaelic Athletic Association (GAA) club athletes, Courtney et al. (49) identified 38.5% (*n* = 47) at risk of LEA, while Pai et al. (113) found that 53% of New Zealand team sport athletes aged >16 years were similarly at risk.

#### 3.1.4 Low energy availability in collegiate athletes

Additionally, among college and university female athletes, LEA is highly prevalent across a variety of sports and competitive levels (137). Torres-McGehee et al. (157) reported that 81% of 121 collegiate female athletes and performing artists (19.8 ± 2.0 years) exhibited LEA, with softball players showing the highest concurrent risk of eating disorder (ED) (**82.4%**), followed by ballet dancers (76%). Scheid et al. (129) found that 68.7% of collegiate athletes were at risk of low energy availability based on LEAF-Q scores. In contrast, Edama et al. (62) observed a lower prevalence of 3.4% (4/116) among female college athletes competing at the national level. Uriegas et al. (161) reported that among female student-athletes attending historically Black Colleges and Universities (HBCUs), 92.6% (*n* = 25) of 27 participants had LEA, with a mean energy availability of 15.9 ± 10.1 kcal/kg FFM/day, highlighting the elevated risk of both LEA and EDs in this population.

#### 3.1.5 Low energy availability in aesthetic and recreational sports

Nonetheless, low energy availability (LEA) is prevalent among female athletes across aesthetic, combat, and recreational sports, as consistently reported in the literature. Athletes participating in aesthetic sports appear to be particularly vulnerable to LEA. Hoch et al. (80) reported low/negative energy availability in 77% professional ballet dancers (*n* = 17) and Meng et al. (102) reported that 41.6% of 166 Chinese elite and recreational athletes across six aesthetic sports (e.g., rhythmic gymnastics, dance sport, and cheerleading) were at increased risk. In a large sample of competitive and recreationally active females, Goldenstein et al. (21) reported that almost half the total participants (631; age: 25 ± 7 years) were at risk for LEA (45%). Gimunova et al. (74) observed a similar high prevalence with 50% of the Recreational female athletes (n = 24, age: 23.71 ± 2.94, Tier I) being at risk of LEA. Silva and Paiva (133) also observed LEA-related indicators among rhythmic gymnasts. Also, Smith et al. (137) demonstrated a risk of LEA in all cheerleaders for the days they participated in cheerleading practice, specifically, 52.6% were at risk of LEA along with ED risk while 47.4% demonstrated LEA without ED risk.

In combat sports (e.g., judo, freestyle wrestling, and sanda), athletes demonstrated further substantial risk. Thomas et al. (150) assumed that athletes who adopt severe weight reduction habits appear to be at higher risk of developing the Triad. Liang et al. (96) reported that 45.2% of Chinese female combat sport athletes were at risk of low energy availability, based on a sample of 84 participants. Likewise, De Maria and Claudia Ridel Juzwiak (55) observed the LEA risk (LEAF-Q > 8) 34% of the participants competing in crossFit, endurance, aesthetic, combat, and team sports with combat athletes presenting the highest risk.

#### 3.1.6 Consequences

LEA may lead to serious consequences that could increase the risk of negative impact on physiological, physical, and overall health, compromising sports performance (171–173). Low energy availability has widespread physiological consequences across female athletes, adversely affecting performance, metabolic, reproductive, and skeletal health. Ackerman et al. (24) reported that athletes with low EA were more likely to experience menstrual dysfunction, compromised bone health, metabolic disturbances, psychological disorders, cardiovascular impairment, gastrointestinal dysfunction, and decreased performance outcomes, including coordination, endurance, and concentration. Consistently, LEA increased the risk of self-reported physiological symptoms and impaired performance as reported by (85). Among gymnasts, especially younger athletes, LEA failed to meet the physiological and training demands (133). LEA has been identified as a predictor of illness contributing to sports incapacity (61).

In addition to systematic health effects, LEA has been extensively studied for its cardiometabolic and performance-related consequences across a wide range of sports and competitive levels (24, 124, 171). LEA disrupts metabolic efficiency and endocrine regulation, which suppresses RMS, changes lipid profiles, and increases cortisol responses (174, 175). These changes compromise cardiovascular function, immune response, and skeletal muscle adaptation, ultimately impairing athletic performance (174, 175). Liang et al. (96) noted that female combat athletes with LEA had significantly reduced VO2 and RMR compared to four weeks prior to competition, alongside decreases in thyroid function. Conversely, REED et al. (120) observed that lower EA during the preseason testing was associated with a higher VO_2max_ in female soccer players. Among endurance athletes, low or reduced EA has been associated with decreased RMR, hypoglycemia, hypotension and hypercholesterolemia, and other clinical features beyond the triad (101). Similarly, LEA was associated with an increased level of low-density lipoprotein (LDL) cholesterol in lean sports (134). Experimental evidence further supports that 14 days of LEA increased cortisol levels in female endurance athletes and exhibited a pronounced impact on the immune system, including altered inflammatory proteome, impaired leukocyte mobilization, and increased capacity for ROS reducing exercise performance (82).

Nonetheless, LEA further impacts reproductive health and menstrual function. Miyamoto et al. (105) in a longitudinal study of 16 elite female rowing competitors demonstrated that a significant increase in EA (*P* < 0.01), alongside adequate carbohydrate intake, were associated with improvement in menstrual function. Consistently, De Maria and Claudia Ridel Juzwiak (55) reported associations between LEA and gastrointestinal and menstrual dysfunctions in Brazilian female athletes. Schaal et al. (128) highlighted that a non-functionally overreached runner who failed to increase energy intake during training overload showed suppressed EA and ovarian function, whereas those adapting positively maintained baseline EA and ovarian function. Miralles-Amorós et al. (104) reported LEA in all professional female handball players and all had eumenorrhea. Moreover, Castellanos-Mendoza et al. (42) reported that competitive Guatemalan racewalkers and runners (*n* = 15) with an EA of <35 kcal·kg FFM^−1^·day^−1^ exhibited ovulatory disturbances, highlighting the impact of EA during the follicular phase on ovulatory status. Courtney et al. (49) found that 34.4% (*n* = 42) adult Irish female in GAA club teams reported a change in menstruation during intervals of increased exercise intensity. LEA exerts a psychological impact as well. Scheid et al. (129) assumed that LEA was associated with increased anxiety in female collegiate athletes.

Bone health is particularly affected by LEA. Holsen Kyte et al. (91) reported that Dual proximal femur BMD positively correlated with estradiol but negatively with LEA symptoms in runners with low BMD restricted to the lumbar spine in those with amenorrhoea. Meng et al. (102) reported that Chinese elite female athletes had higher LEA risk, menstrual disturbances, and primary amenorrhea than recreational athletes, with associated reductions in estradiol and bone mineral density. Gimunova et al. (74) noted that fluctuations in body weight and composition affected postural stability and BMD, but in recreational female athletes, LEA risk scores did not negatively affect these measures.

It is also suggested that female runners with a history of multiple BSIs had greater historical and current energy deficit (70). An increased injury risk was observed among athletes with relative energy deficiency (62). Indeed, Oxfeldt et al. (112) demonstrated that 10 days of LEA impaired daily myofibrillar and sarcoplasmic protein synthesis, reduced lean mass, nitrogen balance, thyroid hormones, and free androgen index, while increasing the cortisol/insulin ratio, thereby negatively effecting skeletal muscle adaptations. Although athletes with LEA had better bone parameters than controls, stress fracture occurred more frequently in the LEA group (4/12 vs. 2/12), highlighting the importance of maintaining adequate EA for skeletal health (68). Taken together, all these findings highlight the widespread and persistent prevalence of LEA across female athletes of all ages, competitive levels and sport disciplines including, team-based, aesthetic, combat, and endurance. LEA in female athletes can compromise skeletal, metabolic, reproductive, cardiovascular, gastrointestinal, psychological, and performance outcomes across diverse sports, and competitive levels. This highlights the critical need for structured and sport-specific screening, early detection, nutritional interventions and tailored preventative strategies to protect athletes at increased risk of experiencing LEA. In sports characterized by extreme training loads, or severe weight reduction practices such as aesthetic, endurance, combat (judo, freestyle wrestling, and sanda), and team sports, more intensive screening approaches are recommended to aid early interventions. This includes monitoring energy intake training load, menstrual function, and bone health (57, 96, 102). Effective preventative strategies include individualized and multidisciplinary combining nutrition plans designed to ensure adequate energy and nutrient availability, adjustments to training volume, and optimize performance (48, 85, 106, 126, 133, 161). Healthcare professionals are encouraged to adopt a multidisciplinary approach that incorporate routine screening, nutrition counseling, and collaboration with coaches and sports dietitians to optimize fueling strategies, support performance, and reduce the risk of inadvertent LEA (14, 171). Furthermore, early education, even among youth athletes, may help prevent the progression of the Triad and REDs, emphasizing that LEA can be mitigated proactively rather than reactively (79, 85, 120).

In conclusion, while low energy availability (LEA) is a physiological consequence of high training volumes, recent evidence suggests a complex and sometimes independent relationship between LEA and Disordered Eating (DE). Notably, a study by Karlsson et al. (84) among recreational runners demonstrated that while symptoms of both EDs and LEA are prevalent (18% and 19% respectively), their coexistence is relatively rare, with only a small fraction of athletes exhibiting both simultaneously (84). This indicates that LEA can occur inadvertently due to a lack of nutritional awareness or high energy expenditure. In contrast, DE may manifest through cognitive dietary restraint—such as shape and weight concerns—without immediately resulting in clinical LEA. This critical divergence necessitates a deeper exploration of the psychopathological drivers of eating behaviors and of whether the energy deficit is a byproduct of accidental under-fueling or a manifestation of intentional restrictive patterns. This exploration is essential for establishing accurate diagnostic and intervention frameworks in the subsequent sections of this review.

### 3.2 Disordered eating and eating disorders

Eating disorders (EDs) are behavioral conditions referred to as psychiatric disorders that some may suffer from as a reaction to facing a certain event or problem, which affects the psychological, physical, and social functions of the patient (2, 176). EDs in women are often associated with disproportionate diets, driven by an obsession with body image (177). In this context, recent research examining the misapplication of pseudo-ketogenic diet model, has revealed that focusing on appearance while neglecting nutritional balance can lead to negative metabolic and immune outcomes, underscoring the risks inherent in misinterpretations of diets (178). EDs in athletes, particularly at the competitive and elite levels, differ from those in the general population. This fact is because they are often linked to athletic performance and championship achievements, rather than solely to physical appearance or weight (179). The statistics regarding the prevalence of EDs among athletes are alarming and align with the prevailing view among many researchers that the sports environment is ideal for concealing eating disorders or unhealthy eating behaviors, such as frequent meal skipping and the use of diuretics, laxatives, or enemas (54, 121, 132, 180). Diagnosing this condition in sports settings is often challenging (179). Determining the prevalence of EDs and classifying risk by age group and type of sport is crucial. A Norwegian study (using clinical interviews) revealed that the percentage of female athletes in leanness sports diagnosed with clinical eating disorders (EDs) was 46.7%, a significantly higher percentage than that recorded in female athletes in non-leanness sports (19.8%) (Supplementary Table S1) (158). This is in line with what other studies have found about running and field sports. For example, 87.5% of female collegiate track and field athletes were found to be at risk for different parts of the Triad, and 73.3% said they had very low energy availability (LEA) (123). While female collegiate athletes generally may exhibit lower levels of body dissatisfaction compared to non-athletes, the risk is particularly concentrated in weight-sensitive sports (25% in weight-sensitive sports vs. 2.9% in non-weight-sensitive sports) (Supplementary Table S1) (121). This prevalence is not limited to elite athletes; in combined endurance sports such as triathlon, 60% of recreational female athletes experienced caloric deficits and macronutrient deficiencies (78), confirming that RED-S is the ultimate outcome of inadequate behaviors across various levels of competition (181). This issue is apparent worldwide, with significant prevalence rates of eating disorders documented in various geographical settings, such as Kenya, Pakistan, and Brazil (Supplementary Table S1) (51, 106, 144).

In a meta-analysis of 56 studies conducted by Chapa et al. (180), the researchers found that the hypothesis suggesting female athletes exhibit higher levels of overall eating disorder psychopathology, drive for thinness, restriction, or loss of control over eating compared to non-athletes was not supported. Chapa et al. (180) revealed two contrasting factors: a protective factor and a risk factor. Female athletes reported significantly lower body dissatisfaction compared to non-athletes (a protective factor attributed to perceived physical function). In contrast, the type of sport was the most significant moderator (a risk factor), with participants in aesthetic/thin sports reporting higher levels of eating disorder psychopathology, while age or competition level did not. Accordingly, it is recommended that prevention and treatment efforts be directed specifically at sports that emphasize shape and weight. Psychological and behavioral factors are central to inadequate dietary behaviors in female athletes and act as a driver of chronic RED-S (32). This obsession translates directly into restrictive behaviors; studies have documented that female athletes with subclinical EDs consume significantly less energy (an average of 1,989 kcal/day) compared to control athletes (Supplementary Table S1) (31). Environmental factors and power dynamics also play a crucial role, with athletic body ideals and coach-athlete power dynamics contributing to an environment that increases the risk of body image disturbance (41), driving female athletes in aesthetic performance sports (such as dancers) to adopt a drive for thinness and cognitive dietary restraint as a coping mechanism (Supplementary Table S1) (122).

Research also reveals a complex interplay between disordered eating behaviors, mental health, and physiological indicators in female athletes. Studies have shown that both EDs and LEA are positively and independently associated with increased anxiety symptoms among collegiate female athletes (Supplementary Table S1) (129). While the relationship between anxiety and EDs may not be evident in all studies, it is recommended to monitor less experienced female athletes who exhibit high anxiety levels, given their increased risk of developing EDs (Supplementary Table S1) (27). Regarding depression, while no evidence was found to suggest that LEA is an independent factor for depression, a strong association was observed between depression and the presence of an ED, suggesting that the relationship between LEA and RED-S syndrome may be primarily mediated by EDs (Supplementary Table S1) (76). Physiologically, these disorders have negative metabolic consequences, with higher eating disorder scores (EDE-QS) being associated with elevated levels of LDL (bad cholesterol), particularly among female athletes participating in weight-sensitive sports (Supplementary Table S1) (134). Disordered eating behaviors (DEAs) have also been linked to an increased risk of primary amenorrhea and dysregulation of stress hormones such as cortisol and prolactin (20). These combined findings confirm that eating disorders remain a widespread behavioral and physiological problem requiring intervention that targets both nutrition and mental health (Supplementary Table S1) (161, 168). Furthermore, physiological evidence indicates that the behavioral dimensions of eating disorders lead to physiological and functional consequences independent of traditional menstrual dysfunction (33, 182). Studies on adolescent runners have shown that cognitive dietary restraint (CDR) is directly associated with reduced energy, carbohydrate, fat, and grain intake (183). Consequently, cognitive restraint (CER) is an independent indicator of internal changes, predicting reduced bone mineral density (BMD) in the spine even after controlling for hormonal factors (39). Intentional weight loss and disordered eating have also been linked to an increased risk of specific musculoskeletal injuries, such as rib pain in lightweight rowers (Supplementary Table S1) (58). Furthermore, the repercussions are not limited to structural injuries but also include an increased risk of other functional health problems, most notably urinary incontinence, which has been significantly associated with disordered eating behaviors (Supplementary Table S1) (93). Therefore, a recent study by Şenol et al. (184) indicated that intuitive eating (IE) supports athletic performance by improving self-regulation, reducing psychological constraints, and enhancing energy availability. IE is an eating approach that encourages developing a healthy relationship with food, promoting positive body image, and focusing on and responding to hunger cues for both physical and mental well-being (185). Accordingly, the results of a study by Thomson et al. (153) confirmed a strong inverse relationship between intuitive eating and levels of eating disorders in female university students, both athletes and non-athletes. The results also showed behavioral differences, with non-athletes scoring higher in unconditional permission to eat, while athletes excelled in aligning food choices with bodily cues, suggesting that intuitive eating provides a behavioral protective factor against disordered eating without necessarily affecting basic physiological parameters in this population (Supplementary Table S1).

Recent research confirms the high prevalence of ED risk and LEA among female athletes. Among female student-athletes at Historically Black Colleges and Universities (HBCUs), a high risk of eating disorders (EDs) was found, reaching 59.3%, with the majority (60%) exhibiting low energy availability (LEA) along with a risk of disordered eating (Supplementary Table S1) (161). This high prevalence is confirmed across diverse athletic communities; among female athletes in Saudi Arabia, the risk of eating disorders was 33.6% (86), and it reached 44% among professional female soccer players (Supplementary Table S1) (126). A study involving adult women engaged in recreational or competitive activity showed that nearly half of the sample were at risk for LEA (45%) and/or ED (45%), regardless of their competitive athletic status (Supplementary Table S1) (21). A similar risk rate was found among Chinese combat sports athletes, with 21.4% of the sample at risk for ED (Supplementary Table S1) (96). This risk is particularly noticeable in sports requiring a specific physique; among elite ballet dancers, a history of eating disorders was found in 47.4% of the group, with a significant statistical association between this history and menstrual cycle disorders (Supplementary Table S1) (95). Managing RED-S requires a multidisciplinary approach, focusing on early intervention and long-term preventative programs. Regarding clinical outcomes, controlled nutritional intervention trials, such as the REFUEL trial, have demonstrated that a modest and safe increase in energy intake does not exacerbate disordered eating or stress but rather improves psychological and nutritional adaptation (Supplementary Table S1) (142). In terms of prevention, intervention programs based on cognitive dissonance theory have shown effectiveness in reducing dietary restriction and internalized thinness ideals in female athletes over the long term (Supplementary Table S1) (34, 141). Given that the behavioral and psychological consequences of disordered eating can persist even after retirement from sport (152), accurate early screening is crucial. In this regard, objective screening tools, such as the Physiologic Screening Test (PST), which incorporates objective measurements, have demonstrated superior sensitivity (87%) and specificity (78%) compared to traditional psychological tests in identifying at-risk athletes (Supplementary Table S1) (54). Furthermore, findings by Mizgier et al. (20). suggest that increased susceptibility to disordered eating behaviors (DEAs), measured by higher EAT-26 scores, is associated with an increased risk of primary dysmenorrhea, along with elevated cortisol and prolactin levels among adolescent basketball players (Supplementary Table S1). To facilitate early detection, screening tools such as the BEDA-Q and LEAF-Q have proven highly effective as reliable screening tools for detecting mild or more severe cases of RED-S, of which eating disorders are a part; the sensitivity of the BEDA-Q for detecting eating disorders reached 71% and its specificity 76% among endurance athletes (Supplementary Table S1) (10). The importance of these tools is particularly evident given that approximately one-third of RED-S cases were detected by BEDA-Q and LEAF-Q combined (10), emphasizing the need to expand awareness and nutritional education related to the Triad/RED-S for all female athletes (21).

### 3.3 Menstrual dysfunction as the core clinical signal of the Female Athlete Triad / RED-S

Across athletic populations, menstrual dysfunction (MD) ranging from subtle menstrual disturbances to oligomenorrhea and amenorrhea represents the most clinically visible endocrine manifestation of chronic low energy availability (LEA) and is therefore central to the identification of the Triad and relative energy deficiency in sport (RED-S) (128, 173). Differences in EA between eumenorrheic athletes and those exhibiting subtle menstrual disturbances highlight the primacy of energy status in menstrual regulation (36) This relationship is further supported by physiologic threshold evidence suggesting that an EA ≥36 kcal·kg FFM−^−1^·day^−1^ is required to maintain normal ovulatory function, indicating a biologically meaningful energetic boundary for reproductive health (42). Importantly, intensified training does not inevitably result in MD; athletes who compensate for increased energy expenditure through *ad libitum* increases in intake are able to preserve baseline EA and ovarian function, underscoring the modifiable nature of this risk when energetic demands are met (128). Consistent with this, adequate carbohydrate intake during observational periods has been associated with the absence of MD, reinforcing the protective role of sufficient dietary intake within the broader framework of EA adequacy (105).

Beyond menstrual outcomes alone, MD is increasingly recognized as a clinically actionable indicator of RED-S–related physiology. Female elite athletes with current MD demonstrate impairments in bone health and endocrine homeostasis, supporting the interpretation of MD as a systemic signal rather than an isolated gynecologic symptom (140). Metabolic adaptations to chronic energy restriction, particularly alterations in resting metabolic rate and energetic efficiency, have also been proposed as complementary physiologic indicators of low EA that may coexist with, precede, or accompany MD, strengthening the rationale for multi-signal screening approaches (60). Notably, MD does not present uniformly across athletic populations. Among NCAA Division I runners, a high burden of menstrual disturbance has been described alongside heterogeneous patterns of RED-S–related consequences, suggesting that MD is a consistent but not exclusive component of broader RED-S phenotypes (40).

#### 3.3.1 Behavioral, psychological, and sport-specific modifiers

Although low EA represents a proximal mechanism, behavioral and psychosocial factors substantially shape MD risk. Disordered eating (DE) is strongly associated with MD, with athletes exhibiting DE more than twice as likely to report oligomenorrhea or amenorrhea compared with those without DE (108). In young competitive distance runners, DE is closely linked to menstrual irregularity, which in turn is associated with reduced bone mineral density (BMD), reinforcing a clinically relevant pathway connecting eating pathology, endocrine disruption, and skeletal vulnerability (45). Similarly, exercising women characterized by high dietary restraint exhibit lower EA and a greater prevalence of MD compared with women with normal restraint, demonstrating that restrictive cognitive–behavioral patterns can translate into endocrine consequences even in the absence of formally diagnosed eating disorders (71). Concordantly, a higher prevalence of severe menstrual disturbances has been reported among exercising women with elevated drive for thinness, a construct itself linked to energy deficiency, reinforcing the behavioral–energetic interface underlying MD risk (73).

Sport-specific context further modifies MD expression. In dancers, a high prevalence of menstrual cycle disorders, delayed menarche, and primary amenorrhea has been reported alongside a substantial history of eating disorders, consistent with prolonged energetic strain in lean-aesthetic sport environments (95). In cheerleaders, self-reported data revealed a high prevalence of Triad components and universal low EA, highlighting that MD-related risk can be widespread within a single sport cohort when energetic imbalance is pervasive (137). Conversely, among elite female athletes in Pakistan, although the risk for DE was substantial, amenorrhea and low BMD were less prominent, suggesting that the expression of MD and Triad components may vary according to population characteristics, sport culture, and nutritional environments (144).

MD may also be preceded by subtler warning signs. Menstrual irregularity has been identified as an early predictor of subsequent menstrual dysfunction, with risk further amplified in lean sports and among athletes who overestimate self-perceived body image, underscoring the importance of early menstrual changes and body-image distortion as upstream indicators (26). Psychological stress may further intersect with menstrual health; elite female paddlers demonstrate elevated risk of anxiety disorders, particularly among less experienced athletes, plausibly reflecting compounded physiological and psychosocial stressors related to training and dual-career demands (27). In parallel, immune-related fluctuations across the menstrual cycle—such as reductions in haptoglobin during the luteal phase and complement component 3 during exercise—illustrate additional physiologic oscillations in athletes, even in the absence of direct associations with appetite-related factors, supporting a systems-level view of MD regulation (145). Moreover, menstrual pain conditions may co-occur with endocrine stress signatures; female basketball players with primary dysmenorrhea exhibit higher prolactin and cortisol concentrations alongside greater susceptibility to disordered eating attitudes, emphasizing the need to interpret menstrual symptoms within broader endocrine–behavioral contexts (20).

#### 3.3.2 Skeletal health, injury risk, and long-term clinical relevance

MD is clinically meaningful due to its frequent alignment with skeletal vulnerability and injury burden, although sport-specific mechanical loading may modify these associations. In female elite athletes, MD has been linked to reduced lumbar spine BMD, supporting its plausibility as a contributor to site-specific skeletal risk (115). Prospective data further indicate that baseline DE predicts future menstrual irregularities and attenuated lumbar spine bone accrual in adolescent endurance runners, highlighting the potential for cumulative endocrine and skeletal consequences during critical developmental windows (30). Injury risk is similarly elevated; among female high school athletes, musculoskeletal injuries have been associated with DE, oligomenorrhea or amenorrhea, and low BMD, situating MD within a broader injury-relevant risk profile (117). In swimmers, menstrual-cycle disorders correlate with injury incidence, while maintenance of appropriate body weight predicts the absence of cycle disturbances, underscoring energetic and anthropometric contributions to menstrual stability and injury risk (169). Nevertheless, some evidence suggests that injury risk may be driven more strongly by DE than by MD alone; among collegiate gymnasts, DE was more strongly associated with nonsurgical time-loss and spine injuries than menstrual irregularity *per se*, emphasizing the importance of evaluating the full Triad constellation (67).

The relationship between MD and bone health is not uniform across sports due to differential mechanical loading. Among dancers with oligo/amenorrhea and apparent undernutrition consistent with Triad syndrome, BMD did not differ from non-exercising controls, and eumenorrheic dancers demonstrated higher BMD, suggesting that intensive weight-bearing activity may partially offset skeletal consequences despite the presence of MD (155). Similarly, former gymnasts exhibit higher adult BMD regardless of prior menstrual interruption, supporting residual skeletal benefits of loading history (87). Comparative analyses between gymnasts and runners further indicate that loading modality exerts a stronger influence on BMD than menstrual status alone (35). In line with this, high-frequency periodized leg resistance training has not been associated with adverse effects on Triad components, indicating that appropriately structured resistance exercise can be compatible with athlete health when energy needs are met (166).

Importantly, the clinical relevance of MD extends beyond sport participation. A history of RED-S has been associated with increased risks of premature labor, preterm delivery, unexplained vaginal bleeding during pregnancy, and lower infant birth weight, extending the implications of MD and energy deficiency into long-term reproductive outcomes (83). From a cardiometabolic perspective, chronic hypoestrogenism in adult female athletes has been described as a potential risk factor for adverse cardiovascular and skeletal biomarker profiles in the absence of appropriate therapeutic intervention (138).

#### 3.3.3 Differential diagnosis and management considerations

Not all menstrual disturbances in athletes reflect low EA. Among female Olympic athletes across diverse sports, most menstrual disturbances have been attributed to polycystic ovary syndrome in the context of anabolic body composition profiles and normal biomarkers of EA, underscoring the necessity of careful differential diagnosis rather than reflexive RED-S attribution (75). Notably, MD does not present uniformly across athletic population. For instance, in Olympic athletes, hyperandrogenism may explain reproductive dysfunction independently of energy status. High school athletes using oral contraceptive pills (OCP) exhibit a higher prevalence of DE compared with non-users, yet the presence of withdrawal bleeding – defined as the exogenous hormone – induced shedding of the uterine lining during the inactive pill phase – can mask underlying menstrual irregularity and increase the risk for musculoskeletal injury (149). Although oral contraceptives may reduce stress fracture risk in some athletes, evidence remains inconclusive, and restoration of normal menses through nutritional and training-load interventions remains a priority in athletes with MD and low bone mass (46).

Intervention studies support nutritional correction as a cornerstone of management. Long-term dietary interventions incorporating modest increases in energy intake alongside dietetic and psychological support have demonstrated increases in body and fat mass without exacerbating disordered eating attitudes, stress, or depressive symptoms in exercising women with oligo/amenorrhea, supporting the feasibility of recovery-focused approaches (142). Broader controlled dietary evidence further indicates that sustained improvements in energy intake and availability are necessary to normalize hormonal profiles and restore menstrual cyclicity (92). Collectively, these findings reinforce the rationale for prioritizing correction of the underlying energy deficit rather than relying on hormonal contraceptives as a default strategy (167).

Finally, because current Triad criteria do not capture all women at risk, alternative frameworks incorporating exercise-related menstrual alterations, disordered eating, and osteopenia have been proposed, reinforcing MD's utility as a screening anchor while acknowledging its limitations as a standalone marker (38). This is particularly relevant given that Triad-related symptoms are not confined to athletes; substantial proportions of sedentary students and high school athletes exhibit one or more Triad components, underscoring the need for broad education and prevention strategies (79). In line with priority prevention, triathletes have been identified as a population at elevated risk for Triad components, emphasizing the importance of education among athletes, coaches, parents, and health professionals (78). Additionally, among Japanese female college athletes, sport intensity and training volume rather than competitive level have emerged as key determinants of Triad risk, highlighting the importance of training-load assessment when MD is reported (127).

Overall, the integrated evidence supports MD as the most actionable clinical entry point for Triad and RED-S detection: it is closely linked to energetic status (36, 42, 60, 105, 128) amplified by behavioral and sport-specific pressures (45, 71, 73, 95, 108, 137, 149), variably expressed through skeletal outcomes modulated by mechanical loading (35, 87, 155, 166), and associated with adverse reproductive and cardiometabolic consequences when unaddressed (83, 138).

### 3.4 Bone mineral density (BMD)

Bone health is a critical component of the Triad and Relative Energy Deficiency in Sport (RED-S), acting as a primary biological endpoint compromised by chronic energy imbalances. The evidence strongly supports a dose–response relationship, in which an accumulation of Triad risk factors is directly linked to a higher likelihood of low BMD in exercising women (28, 72).‏

#### 3.4.1 Etiology of low BMD

The core mechanism underlying compromised BMD is rooted in Low Energy Availability (LEA), in which energy deficiency directly interferes with the anabolic bone effects that exercise would normally promote (68). This negative energy balance is theorized to contribute to measurable bone loss, specifically a loss of lumbar BMD (114), and results in endocrine homeostasis impairment detrimental to skeletal integrity (140).‏

Multiple factors contribute to poor BMD or skeletal pathology. Nutritional and psychological factors like higher cognitive eating restraint (CER) in athletes are identified as an independent predictor of lower BMD (39). Also, screening for indicators such as low ideal body weight is suggested as a useful predictor of low BMD and insufficient trabecular bone microarchitecture in pubescent athletes (81). Duration and Severity of Menstrual Dysfunction influence the bone structure. The severity of bone risk is compounded by duration, as women with a longer history of Exercise-Associated Menstrual Dysfunction (ExMD) demonstrate lower BMD (44). While menstrual irregularity (MI) is strongly associated with low BMD, disordered eating (DE) can independently be associated with low BMD even in the absence of MI (45). The chronic hypoestrogenism resulting from menstrual dysfunction leads to negative changes in BMD and is also suggested to be a risk factor for cardiovascular disease (138). Furthermore, low BMD is associated with low energy expenditure, low body weight, and the Body Pain and Activity score assessed by Questionnaire (BPAQ) (25). Alterations in resting metabolic rate (RMR), indicative of chronic energy restriction, are suggested to flag low energy availability, the precursor to bone compromise (60).

Methodological approaches vary across the literature. Bone health in elite athletes across mixed sports and endurance running was primarily assessed via Dual-energy x-ray Absorptiometry (DEXA) (114, 140, 146). In elite dancers, energetic efficiency was measured via indirect calorimetry, highlighting that lower BMD correlates with menstrual irregularity (60).

#### 3.4.2 Epidemiology and specific risk factors for low BMD

The risk factors for bone compromise are widespread across athletic populations (79). However, certain groups show specific vulnerability regarding bone mineral density outcomes. Some specific populations requiring focused attention include adolescent endurance runners, where poor BMD was independently predicted by factors such as low BMI, low lean tissue mass, menstrual irregularity, and extended duration of endurance running for five or more seasons (29). This emphasizes the risk inherent in sustained, high-volume, low-impact training combined with energy imbalance. Similarly, a direct connection exists between menstrual dysfunction and reduced lumbar spine BMD in female elite athletes, suggesting exercise volume may be a contributing factor to the endocrine disruption (115). In collegiate cheerleaders, all displayed LEA, with high percentages presenting one or two Triad components, placing them at increased risk for future bone consequences (137). Kenyan adolescent participants showed insufficient energy intake and disordered eating, identified as major concerns that threaten long-term bone health (106). In Japanese college athletes, the risk factors for energy imbalance were strongly influenced by sport intensity and training volume (127). In some populations, BMD may be preserved despite risk factors. For instance, in Pakistani elite athletes, the risk for disordered eating was significant, yet low bone mineral density was specifically noted as not being a major concern in that (144). Similarly, female volunteers serving in non-high-income countries experienced higher odds of secondary amenorrhea, a key precursor to bone loss, but their BMD itself was found to be within the expected range, suggesting the full impact may take time to manifest (186).

#### 3.4.3 The link to bone stress injuries (BSI)

The most severe clinical manifestation of low BMD is the increased susceptibility to Bone Stress Injuries (BSIs). Increased Triad Cumulative Risk Assessment (CRA) scores are strongly associated with increased risk for BSIs (103, 146), particularly those affecting trabecular-rich bone (187). Athletes classified into moderate- or high-risk categories based on CRA scores are significantly more likely to subsequently sustain a BSI (147). The risk is substantial; athletes presenting with multiple Triad-related risk factors face a 30%–50% risk of BSI (28), and a history of multiple BSIs is linked to prior low-energy fractures (70). Moreover, musculoskeletal injuries have been explicitly linked to the combination of disordered eating, menstrual dysfunction, and low BMD among high school athletes (117). While clinical eating disorders are uncommon, the high prevalence of athletes “at risk” for an eating disorder places them at increased risk for menstrual irregularity and, consequently, bone injuries (33).

#### 3.4.4 The protective influence of mechanical loading

The nature of mechanical loading is a fundamental determinant of BMD. High-impact and weight-bearing activities, such as gymnastics and volleyball, offer a protective effect, resulting in significantly higher BMD compared to non-loading or low-impact sports like distance running (35, 162). Gymnasts, for example, have exhibited higher total BMD than runners (35), and premenarcheal gymnasts show greater BMD compared to age-matched controls (110). This benefit is often durable, with former gymnasts maintaining higher proximal femur BMD years after reduced physical activity, suggesting a residual effect on adult bone mass (87, 89).

The mechanical benefits may sometimes offset the risk of osteoporosis, as seen in young dancers with oligo/amenorrhoea who did not necessarily present with lower BMD than eumenorrhoeic controls (155). Furthermore, areal BMD Z-score (aBMD-Z) – which represents the number of standard deviations an individual's BMD deviates from the mean of a sex, age, and ethnicity matched population - increases with age in female pre-professional dancers and other female athletes, with a stronger association existing between aBMD-Z and BMI in dancers than in athletes (50). However, this protection is highly site-specific; Norwegian elite runners had higher BMD in the proximal femur and total body but not in the lumbar spine compared with controls (91). Conversely, athletes in low-impact and non-impact sports with accompanying low BMI and oligomenorrhea/amenorrhea are at the highest risk for reduced BMD, underscoring the interplay between energy status and mechanical stimulus (146).

#### 3.4.5 Intervention and clinical management

The most critical intervention focuses on addressing the energy deficit, which is warranted to promote reproductive health and, in turn, address bone health (139). Non-pharmacological nutritional intervention, which prioritizes the correction of the energy deficit to improve energy availability and energy balance while minimizing fat gain, is being validated as a primary treatment approach over the common use of hormonal contraceptives (OCPs) (44, 167). For adolescent athletes, combining acupuncture with weight-bearing exercises has also been explored as an effective non-pharmacological intervention to enhance BMD (23).

While OCPs may reduce the risk of stress fractures in some runners, the evidence remains inconclusive, and clinicians must be vigilant: OCP use can mask menstrual dysfunction, obscuring the persistence of Disordered Eating (DE) and other Triad components that continue to compromise bone health (46, 149).

#### 3.4.6 Screening and awareness

Early identification is key, emphasizing the role of screening tools such as the Low Energy Availability in Females (LEAF) questionnaire (99). However, the current Triad components may not identify all women “at risk” for skeletal compromise, and more appropriate criteria such as exercise-related menstrual alterations, disordered eating, and osteopenia have been suggested (38). Though LEA is highly prevalent among recreational female athletes, specific bone aspects were found not to be directly related to LEA, highlighting the need for nuanced screening that moves beyond single-point assessment (74). Finally, there is a recognized lack of awareness among susceptible groups, such as collegiate cross-country runners, regarding the seriousness of bone compromise and its associated pathology (107). These findings underscore the imperative for multidisciplinary sport science support from a young age, particularly in sports where factors like intentional weight loss (IWL) increase the likelihood of DE, which impacts bone health (58).

#### 3.4.7 Conclusion and future perspectives

In conclusion, this systematic review of 153 studies underscores that the Triad and Relative Energy Deficiency in Sport (RED-S) represent a complex physiological crisis rather than a simple athletic setback. The fundamental driver of these conditions is Low Energy Availability (LEA), which forces the female body into a survival-oriented “starvation mode”. In this state, the metabolism slows significantly to conserve limited energy, systematically deactivating “non-essential” biological systems such as reproduction and skeletal growth to prioritize immediate vital functions.

The evidence synthesized highlights three critical pillars of concern:

- The Metabolic Mask: A healthy body weight or BMI can be deceptive, as metabolic impairments—including elevated blood glucose, cholesterol, and liver enzymes—can occur even in athletes who appear physically fit.
- Hormonal and Skeletal Integrity: Menstrual dysfunction is not merely a side effect of training but a clinical warning of hypoestrogenism. This hormonal deficit, compounded by inadequate caloric intake, disrupts bone remodeling and leads to irreversible declines in bone mineral density and recurring stress fractures.
- The Psychological Predictor: Psychobehavioral factors, particularly Body Image Overestimation and rigorous dietary restriction, act as potent triggers for LEA, independent of the athlete's actual physical requirements.

Moving forward, the management of this disorder necessitates a shift from isolated medical interventions toward a multidisciplinary framework. Successful recovery requires the coordinated efforts of healthcare professionals, coaches, and family members, with a primary focus on restoring energy balance, normalizing menstrual function, and improving bone mineral health. Management strategies to address (RED-S) and the Female Athlete Triad focus on restoring energy balance, implementing multi-stage screening, and promoting cultural awareness within sports environments. The primary objective of treatment is to optimize energy availability (EA) through increased energy intake, reduced exercise expenditure, or a combination of both. Healthy EA for exercising women is generally considered to be approximately 45 kcal/kg of fat-free mass (FFM) per day (68, 188); although physiological function may improve at levels above 30 kcal/kg FFM/day, higher intake is often necessary to support optimal bone mineral density (188). Practical approaches for athletes experiencing an energy crisis include gradually increasing daily caloric intake by 300–600 kcal, with some evidence suggesting that smaller increases of 300–350 kcal/day may be sufficient to restore menstrual function (188–190). Emphasis on dietary composition, particularly adequate protein and carbohydrate intake, is important to replenish glycogen stores and support proper hormonal signaling (188, 189). Nutritional support from a registered sports dietitian is recommended to calculate individual EA accurately and develop personalized meal plans that minimize reliance on supplements (188, 190). Early detection of athletes at risk is critical to prevent long-term consequences, including irreversible bone loss (81, 191). Combining validated screening tools, such as the Low Energy Availability in Females Questionnaire (LEAF-Q) and the short-form Eating Disorder Examination Questionnaire (EDE-QS), can help identify both physiological symptoms of low EA and disordered eating behaviors (99, 101, 134). The 12-item EDE-QS provides a practical alternative to the full 28-item EDE-Q, specifically assessing compulsive exercise, a key risk factor not captured by the LEAF-Q (134). Finally, educational programs for athletes, coaches, and parents are essential to increase awareness of the risks associated with inadequate energy intake and to foster a culture that prioritizes health and performance over harmful weight-control practices (37, 140, 188, 189, 192).

While this review establishes the physiological groundwork of the Triad, there is a critical need to explore targeted intervention protocols. Future perspectives should prioritize evaluating specific nutritional strategies, including optimized macronutrient, micronutrient, and supplementation requirements, further the timing of caloric intake to mitigate the starvation response during high-intensity training. Safeguarding the biological capital of the female athlete must remain the ultimate goal, ensuring that the pursuit of performance does not come at the cost of long-term health and structural integrity.
