# Supplementary material for: Protecting women's health in sport: the role of low energy availability in the Female Athlete Triad and RED-S
Source: Front Sports Act Living. 2026 May 20;8:1776533. doi: 10.3389/fspor.2026.1776533 (PMC13230000; doi:10.3389/fspor.2026.1776533)
Supplement: Supplementary file 1 [file Table1.docx]

**Table 1.** Comprehensive synthesis of articles on energy availability, physiological health, and clinical outcomes in female athletes (n = 151), evaluating the interrelationships between low energy availability, disordered eating, menstrual function, and bone mineral density.

| No. | First Author | Year | Nutrition focus area | Study type | Athlete type/ sport | Sample size | Age | Conclusion |
| --- | --- | --- | --- | --- | --- | --- | --- | --- |
| 1 | Abd-Elfattah, Hanaa Mohsen (23) | 2023 | Dietary estrogens  bone mineral density | Randomized controlled trial | Runners | 44 | Estrogen from dietary origin: 16.10±1.01 acupuncture therapy with weight bearing: 15.77±1.03 weight bearing only: 15.50±1.13 | The most successful intervention for safely increasing BMD in the female athlete triad during adolescence was the combination of acupuncture and weight-bearing exercises. |
| 2 | Ackerman, Kathryn E. (24) | 2019 | Low Energy Availability | Cross-sectional | Mixed sport disciplines | 1000 | 15-30 | Numerous health and performance consequences suggested by the RED-S models are strongly correlated with LEA as determined by self-report questionnaires. |
| 3 | Ahmad N, A. (25) | 2018 | Bone Mineral Density | Cross-sectional | Mixed sport disciplines | 85 | 18-30 | Among female athletes, Bone Mineral Density was linked to BPAQ score, body weight, and energy expenditure. |
| 4 | Amoruso, Irene (26) | 2024 | Female athlete triad  menstrual dysfunction | Cross-sectional | Active female athletes | 288 | 15-40 | In female athletes, irregular menstruation may be an early indicator of menstrual dysfunction. |
| 5 | Aquino Linares, Nieves (27) | 2024 | Eating disorder Menstrual disturbances | Cross-sectional | Elite paddlers | 47 | Group 1= 24.91 group 2= 22.83 | Anxiety disorders are a risk factor for elite Spanish female paddlers. The least experienced athletes are more likely to experience these disorders, most likely as a result of their dual careers. |
| 6 | Barrack M. (28) | 2014 | Female Athlete Triad bone stress injury | Cohort study | Physically active girls and women | 259 | 18.1 ± 0.3 | Among female adolescents and young adults engaged in competitive or recreational exercise, the risk of bone stress injuries (bsis) rose from approximately 15–20% with individual risk factors to 30–50% when multiple female athlete triad–related risk factors were present. |
| 7 | Barrack M. (29) | 2008 | Bone Mineral Density Eating Disorder | Cross-sectional | Runners | 93 | 16.1 ± 0.1 | BMI, lean tissue mass, MI, and endurance running for five or more seasons were all independent predictors of poor BMD, according to multiple regression analysis. There may be a higher risk of poor bone mass in female adolescent endurance runners. |
| 8 | Barrack, Michelle T. (30) | 2021 | Disordered eating menstrual irregularity | Cohort study | Endurance runners | 40 | 15.9 ± 1.0 | In female adolescent endurance runners, there are correlations between baseline disordered eating and subsequent irregular menstruation or decreased lumbar spine bone mass accumulation. |
| 9 | Beals K. (31) | 1998 | Eating disorders | Cross-sectional | Mixed sport disciplines | 48 | Athletes: 27±5 control: 28±5 | Micronutrient status seems to be mostly unchanged, likely as a result of supplement use, even though female athletes with subclinical eating disorders had dietary intakes of energy, protein, carbohydrate, and several micronutrients that were below recommended values. |
| 10 | Beals, K. (32) | 2000 | Eating disorder | Cross-sectional | Mixed sport disciplines | 48 | 26.9 ± 5.1 | Recognizing the physical, psychological, and behavioral characteristics of female athletes may enable earlier identification and management of disordered eating, potentially preventing progression to more severe clinical eating disorders. |
| 11 | Beals, K. A. (33) | 2002 | Disorder eating  Menstrual irregularity bone health | Cross-sectional | Mixed sport disciplines | 425 | \ | Although clinical eating disorders are uncommon among female collegiate athletes, many are "at risk" for eating disorders, which puts them at higher risk for bone injuries and irregular menstruation. |
| 12 | Becker C. (34) | 2012 | Eating disorder | Randomized controlled trial | Mixed sport disciplines | 168 | 18–22 | Showed that at six weeks, both interventions decreased thin-ideal internalization, dietary restriction, bulimic pathology, shape and weight concern, and negative affect; at one year, they decreased bulimic pathology, shape concern, and negative affect. |
| 13 | Bemben D. (35) | 2004 | Bone Mineral Density menstrual status | Cross-sectional | Gymnasts and cross-country runners | 26 | \ | Gymnasts exhibited significantly higher bone mineral density (BMD) than runners (p < 0.05), indicating that BMD is influenced by the nature of mechanical loading. Menstrual status did not have a significant impact on BMD among these female athletes. |
| 14 | Bingzheng, Zhou (36) | 2024 | Energy availability  menstrual disorders | Cross-sectional | Soccer, basketball, volleyball, and track teams | 56 | ≥ 18 | EA varied between female collegiate athletes with SMD and eumenorrhea. The incidence of SMD was negatively connected with EA. |
| 15 | Brown, Katie N. (37) | 2020 | Female Athlete Triad knowledge | Non-randomized controlled trial | Dancers | 24 | \ | In order to promote the general health and wellness of collegiate dancers, triad education ought to be an ongoing part of their curricula. The triad video can be a useful educational tool to support that objective. |
| 16 | Burrows, M. (38) | 2007 | Menstrual dysfunction bone mineral density disordered eating | Cross-sectional | Physically active females | 82 | 31.1±6.7 | More suitable criteria, such as exercise-related menstrual changes, disordered eating, and osteopaenia, are proposed because the current triad components do not identify all women "at risk." |
| 17 | Cano Sokoloff (39) | 2015 | Bone Mineral Density eating disorder | Cross-sectional | Active female athletes | 184 | 14-25 | Lower BMD is independently predicted by higher cognitive eating restraint (CER) in athletes. |
| 18 | Carson, Traci Lyn (40) | 2022 | Menstrual disturbance | Cross-sectional | Runners | 211 | 21.33 ±1.25 | This study found three distinct signs of RED-S consequences and a high burden of menstrual disturbance among NCAA D1 cross country runners. |
| 19 | Carson, Traci Lyn (41) | 2021 | Disordered eating | Qualitative research study | Distance runners | 29 | 18–36 | Female athletes may be more susceptible to disordered eating and body image disorders due to sport body image ideals and the power dynamic between coach and athlete. |
| 20 | Castellanos-Mendoza, M. Carolina (42) | 2023 | Low Energy availability  Menstrual dysfunction | Cross-sectional | Racewalkers and runners | 15 | 14–41 | According to the results of the linear regression analysis, "normal ovulation" requires an energy availability of at least (36 kcal·kg FFM−1·day−1). |
| 21 | Çetiner-Okşin, Bengisu (43) | 2023 | Energy balance Energy availability | Cross-sectional | Basketball players | 15 | 19.53 ± 1.3 | Insufficient consumption of CHO may contribute to the negative energy balance experienced by female basketball players during the training phase. |
| 22 | Cialdella-Kam, L. (44) | 2014 | Low energy availability Bone Mineral Density | Non-randomized controlled trial | Endurance trained women | 17 | Eumenorrheic Controls: 23.1 ± 4.3 Women with exmd: 22.6 ± 3.3 | An effective non-pharmacological treatment for exmd might be a dietary innovation that increases EA and EB while reducing fat gain. |
| 23 | Cobb, K. L. (45) | 2003 | Disordered eating, menstrual irregularity Bone Mineral Density | Cross-sectional | Distance runners | 91 | 18-26 | Menstrual irregularity is strongly correlated with disordered eating in young competitive female distance runners; menstrual irregularity is correlated with low BMD; and disordered eating is correlated with low BMD when menstrual irregularity is absent. |
| 24 | Cobb, K. L. (46) | 2007 | Bone Mineral Density menstrual dysfunction | Randomized controlled trial | Runners | 150 | 18-26 | For female runners, oral contraceptives may lower the risk of stress fractures. It is recommended that oligo/amenorrheic athletes with low bone mass increase their Ca intake and take action to resume regular menstruation, including gaining weight. |
| 25 | Coelho, G. M. (47) | 2013 | Disordered eating | Cross-sectional | Tennis players | 45 | 14.77± 2.16 | Tennis players should be closely watched to prevent harm to their health and performance because they seem to exhibit more severe disorders than controls. |
| 26 | Condo, Dominique (48) | 2019 | Nutritional intake Low Energy Availability | Cross-sectional | Football players | 30 | 18-35 | The female Australian rules football players consume insufficient amounts of calcium and carbohydrates and have poor understanding of sports nutrition. |
| 27 | Courtney, Aoife (49) | 2025 | Low energy availability | Cross-sectional | Gaelic game athletes | 122 | 22 | The high prevalence of elevated LEA risk among female GAA athletes who are unaware of this consequence highlights the significance of education, resource access, prevention, and early detection |
| 28 | Critchley, Meghan L. (50) | 2024 | Bone Mineral Density | Cross-sectional | Pre-professional dancers | 269 | Dancers: 17.6 ± 3.2 Athletes: 22.8 ± 2.6 | In female athletes and pre-professional dancers, abmd-Z rises with age. Dancers have a higher correlation between abmd-Z and BMI than do athletes. |
| 29 | Da Costa, N. F. (51) | 2013 | Disordered eating | Cross-sectional | Swimmers | 77 | 19-Nov | When compared to DE-negative athletes, DE-positive athletes had higher body fat and fat mass percentages, lower protein consumption in the 11–14 age group, and inadequate calcium intake in the 15–19 age group. |
| 30 | Dasa, Marcus Smavik (52) | 2024 | Low energy availability | Cross-sectional | Football players | 60 | 22.5 ± 3.7 | A significant number of the players displayed both primary and secondary indicators, with the observed prevalence of reds indicators ranging from low to extremely high. |
| 31 | Dasa, Marcus Smavik (53) | 2023 | Low energy availability | Cross-sectional | Football players | 60 | 22.5 | The results refute the use of the LEAF-Q to identify Triad conditions and LEA in female football players. |
| 32 | David R. Black (54) | 2003 | Disordered eating Eating disorders | Cross-sectional | Division I, club, and dance team | 148 | 18-25 | Because the Physiologic Screening Test was created especially for female athletes, it appears to be an appropriate replacement for current tests. |
| 33 | De Maria, Uyara Pereira (55) | 2021 | Low energy availability | Cross-sectional | Crossfit, endurance, aesthetic, combat, and team sports athletes | 127 | 18–39 | Brazilian athletes participating in a variety of sports were found to be at risk for triad; however, athletes who are sensitive to weight, particularly combat athletes, are more susceptible. |
| 34 | De Souza, Mary Jane (56) | 2022 | Female Athlete Triad Energy intake | Randomized controlled trial | Exercising women | 40 | 21.3 ± 0.5 | The twelve months intervention may have been too short, and the increase in energy intake (approximately 352 kcal/d) was not enough to improve abmd or increase estrogen, even though it was enough to increase menstrual frequency. |
| 35 | Dervish, R. A. (57) | 2023 | Low energy availability Eating disorders Disordered eating | Cross-sectional | Endurance runners | 524 | 18-24 | Compared to other age groups, a higher proportion of female endurance runners between the ages of 18 and 24 were more likely to experience eating disorder, disordered eating, and LEA. |
| 36 | Dimitriou L. (58) | 2014 | Bone Mineral Density eating disorder | Longitudinal Cross-sectional | Lightweight rowers | 21 | 28.7±4.3 | The findings indicate that early exposure to high-level training and IWL raises the risk of DE and that these athletes may not receive adequate nutritional support. |
| 37 | Dobrowolski, Hubert (59) | 2020 | Low energy availability | Cross-sectional | Soccer players | 31 | \ | The great majority of the group under study showed LEA, which could have detrimental effects on their health or limit their ability to exercise. |
| 38 | Doyle-Lucas (60) | 2010 | Menstrual Irregularity Bone Mineral Density low energy availability | Cross-sectional | Ballet dancers | 30 | Dancers: 24.3 ± 1.3 controls: 23.7 ± 0.9 | These results shed light on the metabolic effects of long-term energy restriction and imply that changes in RMR, or energetic efficiency, could be a sign of low energy availability. |
| 39 | Drew, M. (61) | 2017 | Low energy availability | Cross-sectional | Mixed sport disciplines | 55 | 25.5 ± 7.4 | Low energy availability stands out as the main correlation with illness and has high attributable fractions in the population. |
| 40 | Edama, Mutsuaki (62) | 2021 | Low energy availability | Cross-sectional | Swimmers, long-distance athletics | 116 | 19.8 ± 1.3 | In terms of the number of injured athletes by risk category, there were noticeably more athletes in the injury group than in the non-injury group in the moderate and high-risk categories. |
| 41 | Fahrenholtz, Ida Lysdahl (63) | 2023 | RED-S FUEL intervention | Non-randomized controlled trial | Endurance athletes | 46 | Intervention: 24.1 ± 4.7 control: 25.3 ± 4.8 | In female endurance athletes with symptoms of reds, the FUEL intervention enhanced knowledge about sports nutrition and suggested weak evidence for improved sports nutrition behavior. |
| 42 | Fahrenholtz, Ida Lysdahl (64) | 2023 | RED-S FUEL intervention | Randomized controlled trial | Endurance athletes | 45 | Intervention: 25.2 ± 4.09 control: 24.1 ± 4.7 | Menstrual function and other RED-related symptoms will improve over time if you take part in the FUEL intervention. |
| 43 | Finn, Erin E. (65) | 2021 | Female Athlete Triad Iron status | Cross-sectional | Mixed sport disciplines | 239 | 19.9 ± 1.2 | Triad risk factors were linked to markers for low iron status. |
| 44 | Folscher, L. L. (66) | 2015 | Low Energy Availability disordered eating | Cross-sectional | Comrades Marathon runners | 306 | 39.45±7.97 | Even though 44.1% of the female Comrades Marathon runners being at high risk for the triad, only 7.5% of them were aware of it. |
| 45 | Fryar, Caroline M. (67) | 2023 | Female Athlete Triad  Eating disorders  menstrual irregularity | Retrospective case-control | Former gymnasts | 470 | \ | Compared to those who had irregular menstruation, college gymnasts with disordered eating were more likely to sustain a spine injury and a nonsurgical time loss injury. |
| 46 | Gama, E. M.F. (68) | 2022 | Low Energy Availability bone mineral density | Cross-sectional | Long-distance triathletes | 40 | 36.9 | The effects of exercise on bone are impeded by a lack of energy. |
| 47 | Garay, Jessica L. (69) | 2025 | Low energy availability | Cross-sectional | NCAA division 1 athletics team or highly physically active female | 77 | \ | Female college athletes and other physically active students showed less-than-ideal energy availability. Compared to low energy availability, low RMR ratio seemed to be a more sensitive indicator of reds risk. |
| 48 | Gehman, Sarah E. (70) | 2022 | Low energy availability bone health | Cross-sectional | Runners | 51 | 18-36 | In comparison to controls, women with a history of multiple bsis had more low-energy fractures in the past and had higher estimates of energy deficit both historically and currently. |
| 49 | Gibbs J. (71) | 2013 | Menstrual disturbances Energy availability | Cross-sectional | Exercising women | 86 | Normal DR: 23 ± 4 high DR: 21.9 ± 3.4 | In comparison to women with normal DR, exercising women with high DR showed lower EA and a higher frequency of MD (clinical and subclinical). However, among women who exercised, low EA was not linked to high DR. |
| 50 | GIBBS J. (72) | 2014 | Bone Mineral Density | Etrospective cross-sectional | Exercising women | 437 | 18.0 ± 3.5 | An accumulation of the Triad risk factors was linked to a higher likelihood of low bone mineral density (BMD), indicating a dose–response relationship between the number of Triad risk factors and BMD among exercising women. |
| 51 | Gibbs J. (73) | 2011 | Eating Disorder Energy deficiency menstrual disturbances | Cross-sectional | Exercising women | 117 | 22.9 ± 4.3 | Finding a higher prevalence of severe menstrual disturbances in exercising women with high DT ( drive for thinness), the current study also confirms the link between a high DT score and energy deficiency in exercising women. Conversely, exercising women with normal DT showed a higher prevalence of eumenorrheic ovulatory cycles. |
| 52 | Gimunová, Marta (74) | 2024 | Low energy availability Bone Mineral Density | Cross-sectional | Recreational athletes | 24 | 23.71 ± 2.94 | Among recreational female athletes, LEA is a common occurrence that appears to be influenced by training volume. While the incidence of injuries and reproductive aspects were associated with LEA, bone aspects and postural stability were not. |
| 53 | Goldenstein, Samantha J. (21) | 2025 | Disordered Eating  Low Energy Availability | Cross-sectional | Physically active, premenopausal females | 631 | 25 ± 7 | In addition to a relatively high prevalence of low energy availability and disordered eating risk among physically active females, regardless of athletic status, there is a dearth of information regarding the triad. |
| 54 | HAGMAR M. (75) | 2009 | Energy deficiency Menstrual dysfunction | Cross-sectional | Mixed sport disciplines | 19 | 24.0 ± 3.6 | Female Olympic athletes across various sports exhibited anabolic body composition profiles and normal biomarkers of energy availability. Most menstrual disturbances identified were attributed to polycystic ovary syndrome (PCOS). |
| 55 | Halioua, Robin (76) | 2024 | Low energy availability Eating disorder | Cross-sectional | Weight-sensitive sports | 57 | 18–39 | There is no proof that LEA causes depression in female athletes on its own. It seems to be mostly associated with LEA and reds when DE/ED is present. |
| 56 | Hasdemir P. (77) | 2016 | Female athlete triad  menstrual irregularity | Cross-sectional | Mixed sport disciplines | 172 | \ | Excessive participation in sports can be dangerous for young women. There may be a connection between irregular menstruation and lower BMI. |
| 57 | Hoch A. (78) | 2007 | Disordered eating menstrual status | Cross-sectional | Club triathlon team | 15 | 35 ± 6 | Triathletes are at risk for elements of the female athlete triad. |
| 58 | Hoch A. (79) | 2009 | Low energy availability  menstrual dysfunction bone mineral density | Cross-sectional | Mixed sport disciplines | 160 | Athletes: 16.53 ± 0.95 controls: 16.46 ± 1.17 | A significant portion of sedentary students (65%) and high school athletes (78%) experience one or more of the triad's symptoms. Both girls and women who are physically active and those who lack exercise face serious health risks from the triad or any of its elements. |
| 59 | Hoch, A. Z. (80) | 2011 | Female athlete triad | Cross-sectional | Dancers | 22 | 23.2 ± 4.7 | Reduced BMD, irregular menstruation, and low serum estrogen were all associated with endothelial dysfunction. |
| 60 | Ikegami, Nodoka (81) | 2022 | Low Energy Availability Bone Mineral Density | Cross-sectional | Gymnasts, track and field athletes | 21 | 12−15 | In pubescent female athletes, screening for low ideal body weight may be a helpful indicator of low BMD and inadequate trabecular bone microarchitecture. |
| 61 | Jeppesen, Jan Sommer (82) | 2024 | Low energy availability | Randomized Controlled Trial (single-blinded) | Endurance athletes | 12 | 26.8 ± 3.4 | In female athletes, 14 days of LEA raised cortisol levels and had a significant impact on the immune system, including enhanced ROS production capacity, modified plasma inflammatory proteome, and decreased exercise-induced leukocyte mobilization. |
| 62 | Jones, Paris A.T. (83) | 2025 | RED-S Menstrual dysfunction | Cross-sectional | Mixed-sport female athletes | 1025 | 33.10 ± 3.43 | The history of reds is linked to noticeably lighter babies and raises the risk of early labor, pre-term delivery, and unexplained vaginal bleeding during pregnancy. |
| 63 | Karlsson, Elin (84) | 2023 | Low energy availability  Eating disorders | Cross-sectional | Runners | 89 | 18-39 | The findings show that adult females at all athletic levels, including recreational ones, frequently experience symptoms of eds and LEA. |
| 64 | Kettunen, Oona (85) | 2023 | Low energy availability | Observational longitudinal study | XC skiers and biathletes | 23 | 17.1 ± 1.0 | Restricting nutritional intake may not be a good strategy to change body composition in young female athletes, as lower Fat% was linked to higher macronutrient intake. Additionally, according to LEAF-Q, lower overall CHO intake and EA raised the risk of LEA. |
| 65 | Khatib, Mai A. (86) | 2024 | Low energy availability  Eating Disorder | Cross-sectional | Mixed sport disciplines | 119 | 21.92 ± 4.54 | By creating educational programs about energy intake and healthy physical activity routines, it is necessary to increase public awareness of the problems of LEA, eating disorders, and exercise addiction and their effects on the body. |
| 66 | Kirchner E. (87) | 1996 | Menstrual history Bone Mineral Density | Cross-sectional | Gymnasts | 33 | \ | Nutrient intakes did not differ between FG and FC, and there were no differences in BMD between FG who consistently had regular menstrual cycles and those who had previously experienced a menstrual cycle interruption (> or = 3 mo). |
| 67 | Koltun, Kristen J. (88) | 2020 | Female Athlete Triad | Cross-sectional | Exercising women | 166 | 21.7±0.3 | Dietary restriction for the low EA risk factor can be objectively identified using disordered eating questionnaires. Delayed menarche can be used in place of low BMD and LEA for oligomenorrhea/amenorrhea when a risk factor cannot be determined. |
| 68 | Kudlac J. (89) | 2004 | Dietary intake Bone Mineral Density | Longitudinal cross-sectional | Gymnasts | 19 | Baseline: 20.4 ± 1.2 Follow-up: 24.2 ± 1.7 | Changes in bone mineral density (BMD) among former gymnasts appear to be site-specific, with gymnasts maintaining higher proximal femur BMD than controls despite reduced physical activity. |
| 69 | Kuikman, Megan A. (90) | 2025 | Low energy availability | Non-randomized controlled trial | Elite race walkers | 20 | 26.5 ± 6.5 | When female endurance athletes lived and trained at altitude, their RMR temporarily increased, but LEA exposure had no effect. It is unlikely that the slight increase in RMR (50–75 kcal·d−1) will have a clinically significant impact on an athlete's overall daily energy needs. |
| 70 | Kyte, Karoline Holsen (91) | 2023 | Bone mineral density  Low energy availability | Cross-sectional | Elite long-distance runners | 30 | 25–30 | When compared to controls, Norwegian female elite runners had higher BMD Z-scores in the dual proximal femur and total body, but there was no difference in the lumbar spine. |
| 71 | Łagowska K. (92) | 2014 | Menstrual irregularities | Non-randomized controlled trial | Rowers, synchronized swimmers, and triathlonists | 31 | 18.1 ± 2.6 | The benefits of an adequate energy intake and availability on hormone concentration, as well as the role of energy deficiency in menstrual disorders among young female athletes, are further supported by this report. |
| 72 | Laino, F. (93) | 2023 | Eating disorder | Case-control | Mixed sport disciplines | 270 | 25.8±8.6 | Risk behaviors for eating disorders were more common among female athletes with UI. |
| 73 | Langa, Dorota(94) | 2025 | Low energy availability | Cross-sectional | Top-performing female triathletes | 20 | 37.8 ± 9 | By lowering the energy density of the diet, foods richer in plant proteins, fiber, and pufan6 may put female triathletes at risk for LEA. |
| 74 | Lehbil, S. (95) | 2025 | Eating disorder Menstrual cycle disorders | Cross-sectional | Elite ballet dancers | 38 | 17–42 | 71% percent of the dancers have experienced cycle disorder at least once. Fifty percent of them had either primary amenorrhea or menarche after the age of fifteen. 47.4% had a history of eating disorders. |
| 75 | Liang, Yiheng (96) | 2025 | Low energy availability Eating disorder | Cross-sectional | Combat athletes (judo, freestyle wrestling, and sanda) | 84 | Elite athletes: 20 ± 3 Recreational athletes: 17 ± 2 | Chinese female combat sport athletes were at risk for LEA, but there was no discernible difference in the prevalence of LEA between elite and recreational athletes. |
| 76 | Lodge, Melissa T. (97) | 2022 | Female Athlete Triad knowledge | Cross-sectional | Cross-country athletes | 275 | 20 ± 1 | Young women who participate in sports excessively may be at risk. There might be a link between lower BMI and irregular menstruation. |
| 77 | Loud K. (98) | 2005 | Dietary intake  disordered eating | Longitudinal cross-sectional | Preadolescent and adolescent actine girls | 5461 | 13.9 | Stress fractures were not independently linked to disordered eating. |
| 78 | Łuszczki, Edyta (99) | 2021 | Low Energy Availability bone mineral density | Cross-sectional | Football players | 34 | 15.41±1.42 | Early detection of Triad/Red-S symptoms using screening instruments like the LEAF questionnaire is crucial for shielding young athletes from long-term harm brought on by the development of risk factors linked to the condition. |
| 79 | Melin A. (100) | 2015 | Low energy availability | Cross-sectional | Elite endurance athletes | 40 | 26.2 ± 5.5 | The significance of energy deficiency prevention, early detection, and treatment is highlighted by the high prevalence of ED, MD, and compromised bone health. |
| 80 | Melin, A. (101) | 2014 | Screening tool for Triad | Cross-sectional | Mixed sport disciplines | 84 | 18-39 | To facilitate early detection and intervention for female athletes at risk for the Triad, the LEAF-Q is a quick and simple screening tool that can be used in conjunction with other validated DE screening tools. |
| 81 | Meng, Kun (102) | 2020 | Low energy availability | Cross-sectional | Mixed sport disciplines | 52 | 20 ± 3 | Chinese female athletes who participate in aesthetic sports are at risk for LEA, and female elite athletes have a much higher prevalence of elevated LEA risk than recreational athletes. |
| 82 | Miller Olson, Emily K. (103) | 2024 | Female Athlete Triad bone-stress injuries | Retrospective cohort | Division I collegiate athletes | 239 | \ | In collegiate athletes, a higher Triad score is linked to a higher risk of bone-stress injuries but not non-BSI. |
| 83 | Miralles-Amorós, Laura (104) | 2023 | Low energy availability | Randomized controlled trial | Professional handball players | 21 | 22 ± 4 | All professional female handball players have been found to have low energy availability. |
| 84 | Miyamoto, Mana (105) | 2021 | Low energy availability  menstrual irregularity | Longitudinal Cross-sectional | Rowing competitors | 16 | 16-18 | Improved menstrual function may result from adequate EA levels and CHO intake. |
| 85 | Mizgier, Małgorzata (20) | 2025 | Disordered eating  Menstrual dysfunction | Cross-sectional | Basketball players | 25 | 16 | Prolactin and cortisol levels are higher in female basketball players with primary dysmenorrhea, and they are more susceptible to disordered eating attitudes. |
| 86 | Muia, E. (106) | 2016 | Energy availability bone mineral density Eating Disorder | Cross-sectional | Runners | 61 | 16–17 | Athletes had lower EA than non-athletes, and more athletes had both clinical menstrual dysfunction and clinically low EA. One or more subclinical and/or clinical Triad components were present in Kenyan teenage participants. |
| 87 | Nguyen V. (107) | 2014 | Bone health | Cross-sectional | Cross-country runners | 65 | NAIA: 20.13 ± 1.46 NCAA Division III: 19.58 ± 1.21 NCAA Division II: 20.70 ±1.16 NCAA Division I: 19.50 ± 1.09 | Women who would be more susceptible to osteoporosis and the Triad, including female collegiate cross-country runners, had no perception of themselves as having the disease or that it would be a serious condition if they were to be diagnosed. |
| 88 | Nichols, J. F. (108) | 2007 | Disordered eating menstrual irregularity | Cross-sectional | Lean-build and nonlean-build sports | 423 | 15.7±1.2 | High school athletes' DE and MI are correlated, showing that LB athletes have higher MI but lower DE than NLB athletes. |
| 89 | Nichols, J. F. (109) | 2006 | Prevalence of Triad Eating Disorder | Cross-sectional | Mixed sport disciplines | 170 | 13-18 | Although the full female athlete triad was low, a sizable portion of the athletes may be at risk for long-term health issues related to irregular menstruation, disordered eating, or low bone mass. |
| 90 | Nickols-Richardson (110) | 2000 | Bone Mineral Density Dietary intake | Cross-sectional | Gymnasts | 32 | Athletes: 10.5 ± 1.5 controls: 10.5 ± 1.3 | Compared to controls who are the same age, height, and weight, premenarcheal gymnasts had a greater BMD. |
| 91 | Oxfeldt, Mikkel (111) | 2024 | Low energy availability | Randomized controlled trial | Trained females | 30 | 18-30 | Ten days of LEA led to decreased muscle glycogen levels and decreased performance (absolute values). These impairments were partially restored after two days of OEA recovery. |
| 92 | Oxfeldt, Mikkel (112) | 2023 | Low energy availability | Randomized controlled trial | Trained females | 30 | LEA: 24 ± 3 optimal EA: 26 ± 3 | When trained females engage in exercise training, LEA reduces the synthesis of myofibrillar and sarcoplasmic muscle proteins. |
| 93 | Pai, Namratha N. (113) | 2024 | Low energy availability nutrition knowledge | Cross-sectional | Team sports | 100 | >16 | More nutrition education is required for team sports athletes, as evidenced by their low nutrition knowledge and high rates of individuals "at risk" of LEA. |
| 94 | Pollock N. (114) | 2010 | Female athlete triad Bone Mineral Density | Longitudinal cross-sectional | Elite endurance runners | 44 | 22.9 ± 6.0 | The idea that negative energy balance contributes to bone loss in athletes may be supported by the correlation between increased training volume, a tendency toward menstrual dysfunction, and an increase in lumbar BMD loss. |
| 95 | Punpilai S. (115) | 2005 | Menstrual status Bone Mineral Density | Cross-sectional | Mixed sport disciplines | 63 | 17.8 ± 1.8 | There may be a connection between menstruation dysfunction and lumbar spine BMD, and exercise may be a contributing factor in menstrual dysfunction in female elite athletes. |
| 96 | Quah, Y. V. (116) | 2009 | Prevalence of Triad | Cross-sectional | Mixed sport disciplines | 67 | 13-30 | Among Malaysian female elite athletes, the overall prevalence of two or three of the female athlete triad's conditions is comparatively low. On the other hand, eating disorders were very common (89.2%). |
| 97 | Rauh M. (117) | 2010 | Disordered eating menstrual dysfunction bone mineral density | Prospective cohort study | Interscholastic sports | 163 | 15.7 ± 1.3 | Among female high school athletes, musculoskeletal injuries were linked to disordered eating, oligomenorrhea/amenorrhea, and low BMD. |
| 98 | Raymond-Barker, P. (118) | 2007 | Female athlete triad | Cross-sectional | Trampoline gymnasts | 59 | 33.88 ± 9.74 | The lack of a difference in nutrition knowledge between athletes who are "at risk" and those who are "not at risk" indicates that the Triad's restricted eating is not caused by ignorance. |
| 99 | REED J. (119) | 2013 | Low energy availability | Cross-sectional | Division I female soccer players | 19 | \ | During the middle of the season, body dissatisfaction and the desire to be thin were inversely correlated with energy availability. Despite this, the majority of Division I female soccer players are not susceptible to low energy availability. |
| 100 | Reed J. (120) | 2014 | Low energy availability | Cross-sectional | Soccer players | 19 | 18-21 | The Triad and low EA conditions may be avoided by acknowledging inadequate carbohydrate intake and adopting the practice of eating lower energy-dense meals. |
| 101 | Reinking (121) | 2005 | Disordered eating | Cross-sectional cohort study | Lean and non-lean sports | 146 | Athletes: 19.7 ± 1.1 Nonathletes: 20.2 ± 1.2 | Compared to women who did not play collegiate sports, female athletes did not show increased signs of eating disorder. Our findings, however, indicate that athletes who participate in lean sports are more likely than those who do not suffer from eating disorders. |
| 102 | Robbeson J. (122) | 2015 | Disordered eating  energy status | Cross-sectional | Dancers | 52 | \ | Education on healthy weight management techniques is necessary because female dancers are at risk for DE behavior and many have suboptimal energy status, which may be connected to their desire to look more attractive. |
| 103 | Robbeson, Justine G. (123) | 2019 | BMD, disordered eating, LEA, menstrual dysfunction | Cross-sectional | Track and field athletes | 16 | \ | More than three fourth of these student track and field athletes were categorized with different combinations of the female athlete triad's components, and more than two thirds had poor estimated energy availability. |
| 104 | Rogers, Margot Anne (124) | 2021 | Low energy availability | Cross-sectional | Mixed sport disciplines | 75 | 18-32 | Since there were typically high negative predictive values (range 76.5-100%) for conditions related to LEA, use a screening tool to rule out risk of LEA-related conditions or to create selective low-risk groups that do not require management. |
| 105 | Rogers, Margot Anne (125) | 2021 | Prevalence of Triad | Cross-sectional | Mixed sport disciplines | 112 | 15–32 | The RED-S model's symptoms were common in this cohort, indicating the need for better awareness, monitoring, and treatment of these symptoms in this demographic. |
| 106 | Saifi, Alisha (126) | 2024 | Low energy availability disorder eating | Cross-sectional | Football players | 25 | 19-30 | LEA and mental health conditions like eating disorders and sleep disturbances were more common in female football players. Sleep disturbance and disordered eating were not associated with LEA. |
| 107 | Sawai A. (127) | 2018 | Menstrual status bone health | Cross-sectional | Mixed sport disciplines | 531 | 19.7±0.9 | The key variables influencing Triad risk in Japanese female college athletes are sport intensity and training volume, but not competitive level. |
| 108 | Schaal, Karine (128) | 2021 | Low energy availability  menstrual dysfunction | Longitudinal Cross-sectional | Runners | 16 | 21-33 | In order to maintain baseline EA and ovarian function, runners who positively adapted to training overload (TO) increased their ad libitum energy intake. |
| 109 | Scheid, Jennifer L. (129) | 2024 | Low energy availability  disordered eating | Cross-sectional | Division I, II, or III collegiate athletes | 115 | 19.9 ± 0.1 | The Triad or RED-S risk was linked to worse anxiety symptoms, and more than half of the collegiate female athletes showed moderate to severe anxiety. |
| 110 | Schtscherbyna A. (130) | 2009 | Prevalence of Triad | Cross-sectional | Elite swimmers | 78 | 12.38 ± 0.2 | Many athletes showed signs of partial Triad, particularly disordered eating. |
| 111 | Sharp, Samantha(131) | 2025 | Low energy availability  malnutrition | Cross-sectional | Endurance runners | 70 | 18–55 | Regardless of age, reds are common among female runners. BIA markers of malnutrition, such as FFMI, FMI, and PA, may serve as practical and innovative clinical markers of reds in female runners. |
| 112 | Sharps, Francis Robert Jose (132) | 2022 | Low energy availability Eating disorders Disordered eating | Cross-sectional | Professional, competitive and recreational athletes | 112 | 18-40 | Risk factors for ED/DE and LEA in female athletes may be predicted by age and level of competition. |
| 113 | Silva M. (133) | 2015 | Low energy availability | Cross-sectional | Gymnasts | 67 | 18.7 ± 2.9 | There have been reports of low intakes of calcium, iron, magnesium, vitamins D, E, and K, folate, and pantothenic acid. Vitamins A, B-6, B-12, C, manganese, zinc, thiamine, riboflavin, and niacin intakes were all above-adequate (P < 0.05). |
| 114 | Silvennoinen, Julia I.K. (134) | 2024 | Low energy availability Eating disorder | Cross-sectional | Finnish national- to international-level athletes | 176 | 16–35 | Among athletes participating in lean sports, LEAF-Q and EDE-QS were linked to elevated LDL cholesterol levels. |
| 115 | Sinaga, Evi Susanti (135) | 2023 | Female Athletes Triad knowledge | Descriptive study | Mixed sport disciplines | 61 | 14.74±1.84 | Young female athletes from Papua New Guinea are ignorant of the risk factors and components of Triad. |
| 116 | Skorseth, Paige (136) | 2020 | Prevalence of Female Athlete Triad Iron Supplementation | Cross-sectional | Distance runners | 38 | 16.90 ± 1.00 | High school distance runners had a high prevalence of Triad risk factors. The Triad score, which could be a sign of low energy availability, was inversely correlated with free T3. |
| 117 | Smith, Allison B. (137) | 2022 | Menstrual dysfunction bone mineral density  low energy availability | Cross-sectional | Competitive cheerleaders | 19 | 20.3 ± 1.2 | 52.6% showed two Triad components using self-reported menstrual data, 10.5% showed two Triad components using hormonal evaluations, and 47.7% showed one Triad component. Every cheerleader had LEA. |
| 118 | Soleimany, G. (138) | 2012 | Bone Mineral Density menstrual dysfunction | Cohort study | Mixed sport disciplines | 22 | 23.55± 5.48 | A negative correlation between total cholesterol and VLDL levels and the rate of increase in bone mineral density in the spine region. Each of these factors has a detrimental effect on BMD, which can also affect the lipid profile. |
| 119 | Southmayd, E. A. (139) | 2017 | Energy deficiency bone mineral density | Cross-sectional | Exercising women | 60 | 18–30 | In order to address the distinct contributions of energy status versus estrogen status to bone health, efforts are necessary to correct energy deficiency, which in turn may promote reproductive health. |
| 120 | Stangerup, Ida(140) | 2025 | Bone Mineral Density Menstrual dysfunction | Cross-sectional | Mixed sport disciplines | 17 | 24.8 ± 5.5 | The current data highlight the significance of MD as a potential marker of reds in female elite athletes and show impairment in bone health and endocrine homeostasis in these athletes. |
| 121 | Stewart, Tiffany M. (141) | 2019 | Eating disorders | Cluster randomized controlled trial | Mixed sport disciplines | 481 | 19 | For a number of important eating disorder symptoms and risk factors in female collegiate athletes, the FAB project effectively produced favorable long-term results. |
| 122 | Strock, Nicole C.A. (142) | 2023 | Eating disorders  menstrual disturbances | Randomized controlled trial | Exercising women | 113 | 21.9 ± 0.4 | In exercising women with Oligo/Amen, a long-term nutritional intervention increases body and fat mass without increasing stress, depressive symptoms, or attitudes related to disordered eating. |
| 123 | Suryawati (143) | 2020 | Eating disorders | Cross-sectional | Mixed sport disciplines | 86 | 11_21 | Stress and a poor perception of one's body are risk factors for eating disorders in young female athletes. |
| 124 | Syed, Jaweria (144) | 2022 | Disordered eating menstrual dysfunction bone mineral density | Cross-sectional | Mixed sport disciplines | 60 | 23.57±2.4 | Although amenorrhea and low BMD were not major concerns, the prevalence of disordered eating risk was found to be significant among Pakistani female elite athletes. |
| 125 | Tanabe, Kazuhiro (145) | 2024 | Appetite regulation menstrual cycle | Cross-sectional, pilot study | Softball | 10 | 20.6±0.7 | There is a notable reduction in complement component 3 during exercise and in haptoglobin during the luteal phase. Nevertheless, no correlation was found between these changes and factors related to appetite. |
| 126 | Tenforde, Adam Sebastian (146) | 2018 | Bone mineral density | Cross-sectional | Mixed sport disciplines | 239 | 19.9 ± 1.2 | BMD is influenced by Triad risk factors as well as sport type. Reduced BMD is most likely to occur in athletes who participate in low-impact and non-impact sports, have low BMI, and have oligomenorrhea or amenorrhea. |
| 127 | Tenforde. A. (147) | 2017 | Female Athlete Triad  bone stress injuries (bsis) | Cohort study | Mixed sport disciplines | 323 | 20.0 ± 1.3 | The Female Athlete Triad Cumulative Risk Assessment Score was used to categorize 29% of female collegiate athletes in this study into moderate- or high-risk groups. Athletes at moderate and high risk were more likely to develop a BSI later on. |
| 128 | Thein-Nissenbaum (148) | 2011 | Female athlete triad | Cross-sectional | Aesthetic, endurance, and team/anaerobic | 311 | 15.4 ± 1.2 | Among female athletes in high school, DE and MD are highly prevalent. Additionally, during a sporting season, athletes with DE were more than twice as likely to suffer a sports-related injury. |
| 129 | Thein-Nissenbaum, J. M. (149) | 2014 | Disordered eating menstrual irregularity  bone health | Retrospective cohort | Mixed sport disciplines | 291 | OCP Users: 16.4 ± 1.1 Non-OCP Users: 15.3 ± 1.1 | High school female athletes who use ocps have a higher prevalence of DE than those who do not, even though MI and INJ rates are similar across groups. |
| 130 | Thomas, Sarah (150) | 2021 | Low Energy Availability | Cross-sectional | Combat sport | 102 | 28.8±4.5 | The results of this study show that among female athletes participating in combat sports, more drastic weight-cutting practices may raise the risk of the female athlete triad. |
| 131 | Thompson S. (151) | 2007 | Female Athlete Triad calcium intake | Descriptive study | Runners | 300 | \ | Over 50% of women who reported irregular menstrual cycles did not consume the recommended 1,500 mg/d of calcium, and about 29% of all athletes did not consume enough calcium. |
| 132 | Thompson, Alexandra J. (152) | 2021 | Eating disorders | Longitudinal Cross-sectional | Gymnasts and swimmers | 194 | 25.75 ± 1.19 | After retirement, 51% of athletes who had previously been diagnosed with disordered eating behaviors continued to do so, compared to 23.5% of athletes who had been healthy during their college years. |
| 133 | Thomson, Janie (153) | 2025 | Disordered eating Intuitive eating | Cross-sectional | Runners | 13 | Athletes: 19.5 ± 1.4 non-athletes: 19.9 ± 1.3 | In college-age females, intuitive eating is linked to healthy eating habits and was unrelated to body composition, bone density, or energy availability. |
| 134 | Thralls, K. (154) | 2016 | Female athlete triad | Cross-sectional | Mixed sport disciplines | 320 | 15.9± 1.2 | Adolescents with enstrual dysfunction MD and low BMD may be predicted by low age-adjusted BMI and low IBW, which are evidence-based clinical indicators that can be practically assessed in the field. |
| 135 | To, W. (155) | 2011 | Bone Mineral Density Menstrual irregularity | Cross-sectional | Dancers | 47 | 17–20 | Eumenorrheic dancers actually had higher BMD than non-exercising eumenorrheic controls, but dancers with oligo/amenorrhea and apparent undernutrition that fit the clinical diagnosis of female athlete triad syndrome did not have lower BMD. |
| 136 | Toews, Briana (156) | 2023 | Low energy availability   Nutritional Preparedness | Cross-sectional | Volleyball athletes | 21 | 19-22 | Regardless of their living situation, the results imply that female collegiate volleyball players may have low energy availability. |
| 137 | Torres-mcgehee, Toni Marie (157) | 2021 | Energy Availability  Eating Disorder | Cross-sectional | Soccer, beach volleyball, softball, volleyball, and ballet | 121 | 19.8 ± 2.0 | A significant percentage of female athletes and performers in college were at risk for LEA with an ED risk. |
| 138 | Torstveit M. (158) | 2008 | Disordered eating Eating disorders | Randomized controlled trial | Lean and non-lean sports | 331 | Athletes: 22.2 ± 5.8 controls: 29.6 ± 7.9 | Leanness athletes' menstrual dysfunction, non-leanness athletes' self-reported eds, and controls' self-reported use of harmful weight-control methods were all variables that predicted clinical eds and, therefore, were candidates for reliable screening approaches. |
| 139 | Tosi, Marina (159) | 2019 | Knowledge and prevalence of Triad | Comparative study | Skaters, Dancers, and Runners | 712 | 18-25 | Few athletes were aware that they were at risk for the triad. Compared to runners and figure skaters, dancers were more vulnerable. |
| 140 | Traversa, Claire (160) | 2022 | Energy and dietary intake energy expenditure | Cross-sectional | Varsity rugby union players | 15 | 20.5 ± 0.4 | The players' daily CHO intake recommendations were not fulfilled because they used 6% less energy than they expended, resulting in poor to moderate EA. |
| 141 | Uriegas, Nancy A. (161) | 2024 | Low energy availability  Eating disorder | Cross-sectional | Season sports (i.e., volleyball, soccer, basketball) | 27 | 19 ± 1 | The majority of female student-athletes show symptoms of both EDS and LEA. |
| 142 | Valente-Dos-Santos J. (162) | 2018 | Bone Mineral Density | Cross-sectional | Volleyball players and swimmers | 46 | Swimmers: 15.71±0.93 volleyball players: 16.20±0.77 | When compared to non-loading sports like swimming, young female athletes who engage in high-intensity weight-loading activities like volleyball show somewhat higher levels of BMD at the lower limbs. |
| 143 | Vardar S. (163) | 2005 | Prevalence of Triad | Cross-sectional | Mixed sport disciplines | 224 | 19.5 ± 2.6 | Among young Turkish female athletes, the female athlete triad was present in 1.36% of cases. The triad's components of disordered eating and amenorrhea are far more likely to affect female athletes. |
| 144 | Vardardottir, Birna (135,164) | 2024 | Low energy availability  CHO intake | Cross-sectional | Mixed sport disciplines | 41 | 20.4 | In female athletes, repeated exposure to LEA and LCHO is linked to a number of detrimental effects. |
| 145 | Wasserfurth, Paulina (10) | 2025 | Eating disorder  Low Energy Availability | Cross-sectional | Endurance athletes | 50 | \ | According to the IOC reds CAT2, the LEAF-Q and BEDA-Q are useful screening tools for identifying reds cases with mild or more severe severity/risk among German female endurance athletes. |
| 146 | Whitney, Kristin E. (165) | 2021 | Low energy availability | Cross-sectional | Mixed sport disciplines | 1000 | 15-30 | In every sport category, UI was substantially linked to low EA. |
| 147 | Wikström-frisén L. (166) | 2017 | Menstrual cycle | Randomized controlled trial | Resistance training women | 59 | G1:25.0±4.0 G2: 24.5±2.6 Control: 24.5±3.9 | High-frequency periodized leg resistance training was not linked to adverse effects on any components of the female athlete triad. |
| 148 | Williams, Nancy I. (167) | 2019 | Bone health Menstrual dysfunction | Randomized controlled trial | Exercising women | 55 | 18-35 | The justification is that this study is the first RCT to investigate this non-pharmacological, nutritional intervention as a primary treatment, and its positive results could influence and change clinical practice by confirming the correction of the energy deficit over the widespread use of hormonal contraceptives. |
| 149 | Wilwand, Malorie (168) | 2024 | Low energy availability Disordered Eating | Cross-sectional | Runners | 485 | 18 – 25 | One-third of the young adult female recreational runners reported two or more SF, and more than half reported at least one. Women who reported two or more SF in their lifetime were more likely to self-report eating disorders, and be classified as "at-risk" for LEA. |
| 150 | Witkoś, Joanna (169) | 2022 | Low energy availability Menstrual Cycle Disorders | Cross-sectional | Women practicing swimming | 64 | 24.69 ±2.15 | The absence of menstrual cycle disorders was positively correlated with the participants' correct body weight in most swimming-practicing women. |
| 151 | Witkoś, Joanna (170) | 2022 | Energy Deficiency  Female Athlete Triad | Cross-sectional | National kayaking team | 33 | 20.18 | Early identification of Female Athlete Triad symptoms in a number of young females was made possible by the LEA in Females Questionnaire. |
